# Synthesis and Biological Evaluation of Novel Benodanil-Heterocyclic Carboxamide Hybrids as a Potential Succinate Dehydrogenase Inhibitors

**DOI:** 10.3390/molecules25184291

**Published:** 2020-09-18

**Authors:** Jian Yang, Yongtian Zhao, Jun Wan, Mingfang Jiang, Hong Jin, Ke Tao, Taiping Hou

**Affiliations:** Key Laboratory of Bio-Resource and Eco-Environment of Ministry of Education, College of Life Sciences, Sichuan University, Chengdu 610064, China; YJian_1210@163.com (J.Y.); zyttian@163.com (Y.Z.); junwan88888888@163.com (J.W.); 18080776976@163.com (M.J.); taoke@scu.edu.cn (K.T.); houtplab@scu.edu.cn (T.H.)

**Keywords:** benodanil-heterocyclic carboxamide hybrids, antifungal activity, molecular docking, succinate dehydrogenase inhibitor

## Abstract

In order to discover new antifungal agents, twenty novel benodanil-heterocyclic carboxamide hybrids were designed, synthesized, and characterized by ^1^H NMR and HRMS. In vitro, their antifungal activities against four phytopathogenic fungi were evaluated, as well as some of the target compounds at 50 mg/L demonstrated significant antifungal activities against *Rhizoctonia solani*. Especially, compounds **17** (EC_50_ = 6.32 mg/L) and **18** (EC_50_ = 6.06 mg/L) exhibited good antifungal activities against *R. solani* and were superior to the lead fungicide benodanil (a succinate dehydrogenase inhibitor, SDHI) (EC_50_ = 6.38 mg/L). Furthermore, scanning electron microscopy images showed that the mycelia on treated media with the addition of compound **17** grew abnormally as compared with the negative control with tenuous, wizened, and overlapping colonies, and compounds **17** (IC_50_ = 52.58 mg/L) and **18** (IC_50_ = 56.86 mg/L) showed better inhibition abilities against succinate dehydrogenase (SDH) than benodanil (IC_50_ = 62.02 mg/L). Molecular docking revealed that compound **17** fit in the gap composed of subunit B, C, and D of SDH. Furthermore, it was shown that the main interaction, one hydrogen bond interaction, was observed between compound **17** and the residue C/Trp-73. These studies suggested that compound **17** could act as a potential fungicide to be used for further optimization.

## 1. Introduction

**Figure 1:**
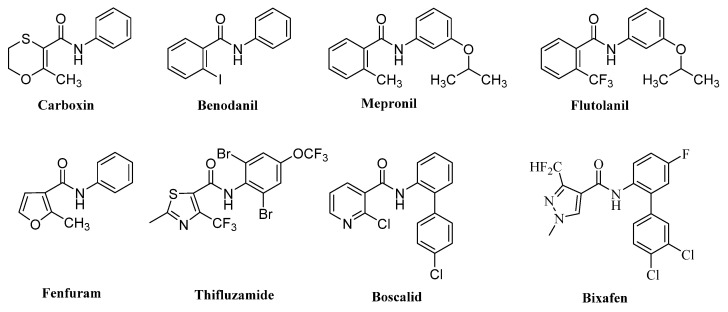
Some commercial aromatic amide fungicides.

Plant diseases always seriously affect production and quality of crops in global agriculture, and thus result in serious economic losses in crop yields and food security [1,2,3]. At present, among the varieties of control methods of plant diseases, chemical controls using fungicides are one of the most effective methods [4,5,6]. As one of the most important categories of agrochemical fungicides, aromatic amide fungicides (a kind of succinate dehydrogenase inhibitors, SDHIs) have been intensively employed all over the world to fight against destructive plant pathogens, such as *Rhizoctonia solani*, *Botrytis cinerea*, and *Sclerotinia sclerotiorum* [7,8,9]. From the first SDHI carboxin to subsequent commercial agrochemical fungicides, they involve benodanil (BASF), mepronil (Kumiai), flutolanil (Nihon Nohyaku), fenfuram (Shell), thifluzamide (Monsanto), boscalid (BASF), bixafen (Bayer), and so on (Figure 1) [5]. However, with the frequent and extensive application of these aromatic amide fungicides, several resistant fungi have been reported [10,11,12]. Therefore, it is necessary to develop new fungicides with novel molecular frameworks to effectively control plant diseases [13,14,15].

**Figure 2:**
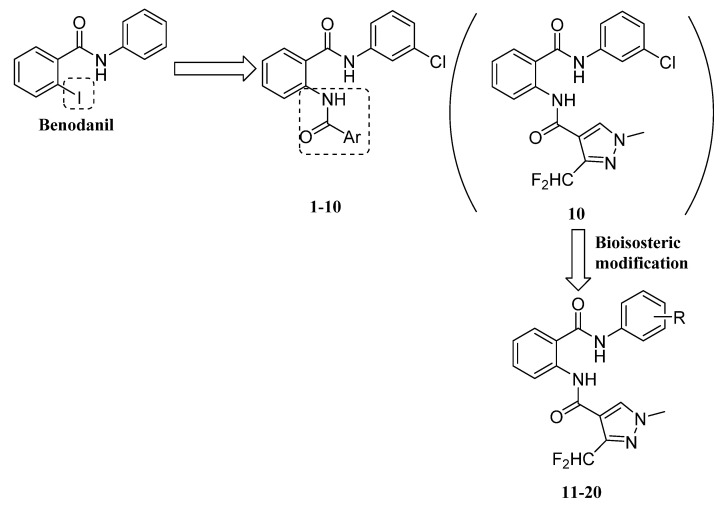
Design strategy of novel benodanil-heterocyclic carboxamide hybrids.

During previous researches for SDHIs, we found that SDHIs all had a common prototypical pharmacophoric scheme, which included a conserved amide function, a structurally diverse carboxyl “core” [16,17,18,19,20,21]. For example, fenfuram has been used as a lead molecule and diarylamines were introduced to design and synthesize a class of fenfuram-diarylamine hybrids with better antifungal activity against *R. solani* than fenfuram [17]. Therefore, in this paper, on the basis of the molecular structure of benodanil, a series of novel benodanil-heterocyclic carboxamides hybrids containing an active skeleton, ortho-replaced benodanil, were designed and the iodine atom on the ortho position of benzene ring was replaced by an aromatic amide group (Figure 2). This modification kept the benodanil scaffold and contained two aromatic amides in the molecular structure of one compound, which was important to the compound’s antifungal activity, and ensured pharmacological properties of benodanil. All of the benodanil-heterocyclic carboxamide hybrids **1**–**20** were identified by ^1^H NMR, ^13^C NMR, and HRMS. In vitro, their antifungal activities were evaluated against four plant pathogenic fungi (*R. solani*, *Fusarium oxysporum*, *Alternaria tenuissima*, and *Alternaria solani*), and enzyme inhibitory, mycelium morphology, and molecular docking were also reported. To the best of our knowledge, this is the first time that the bioactivities of the benodanil-heterocyclic carboxamide hybrids have been studied.

## 2. Results and Discussion

### 2.1. Chemistry

**Scheme 1:**
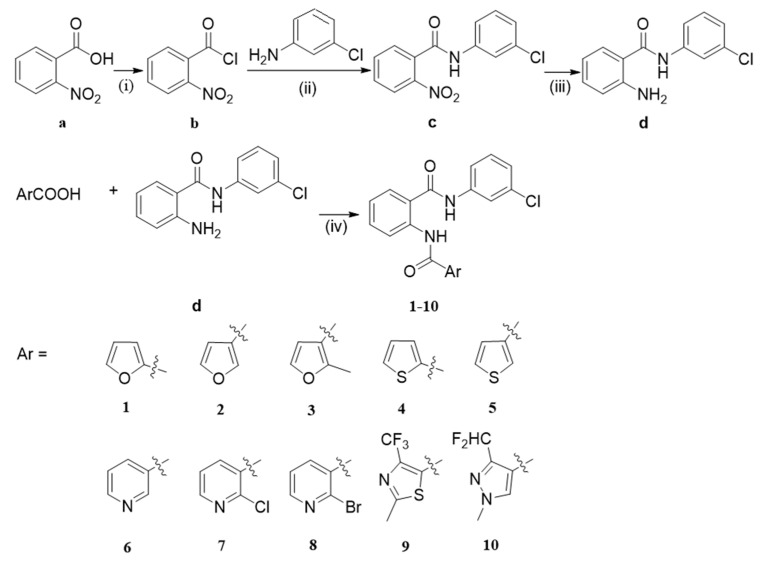
General synthesis of the target compounds **1**–**10**. *Reaction conditions*: (**i**) SOCl_2_/CH_2_Cl_2_, 70 °C, 3 h; (**ii**) CH_2_Cl_2_, Et_3_N, 0 °C r.t., 3 h; (**iii**) Fe/NH_4_Cl, 75 % C_2_H_5_OH, 90 °C 3 h; (**iv**) EDCI/DMAP, DCM, r.t., 6 h.

**Scheme 2:**
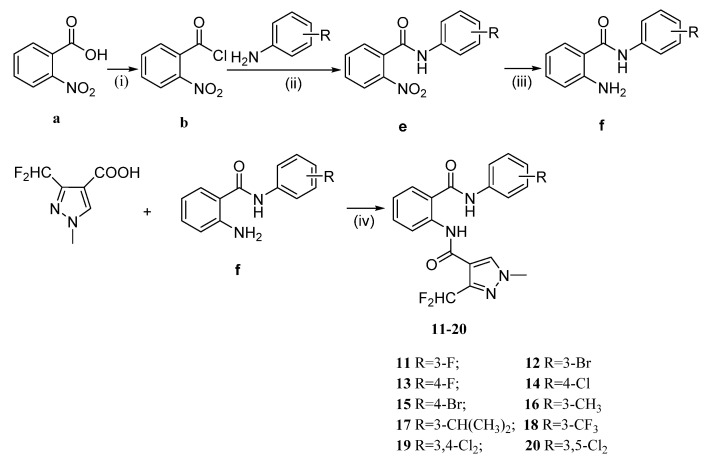
General synthesis of the target compounds **11**–**20**. *Reaction conditions*: (**i**) SOCl_2_/CH_2_Cl_2_, 70 °C, 3 h; (**ii**) CH_2_Cl_2_, Et_3_N, 0 °C r.t., 3 h; (**iii**) Fe/NH_4_Cl, 75 % C_2_H_5_OH, 90 °C, 3 h; (**iv**) EDCI/DMAP, DCM, r.t., 6 h.

Scheme 1 and Scheme 2 and Table 1 and Table 2 summarize the synthesis and chemical structures of the target compounds **1**–**10** and **11**–**20**. The key intermediates **d** (or **f**), shown in Scheme 1 (or 2), were obtained by classic amidation reaction with compound **b** and 3-chloroaniline (or substituted aniline), and reduction with Fe/NH_4_Cl [19,20]. The target compounds **1**–**10** (or **11**–**20**) were obtained, in good yield, by condensation reaction with aromatic acid **d** (or **f**), following reported procedures [21].

### 2.2. Antifungal Activities

The antifungal activities of the synthetic compounds against *R. solani*, *F. oxysporum*, *A. tenuissima*, and *A. solani* were tested, according to our previous reported procedure [21].

Firstly, the results of the in vitro antifungal activities of compounds **1**–**10** at a dosage of 50 mg/L against *R. solani*, *F. oxysporum*, *A. tenuissima*, and *A. solani* were listed in Table 1. Here, their antifungal activities were expressed as the inhibition percentage [21]. Although it seemed impossible to find an obvious structure activity relationship, it was found that compounds **1**–**10** exhibited antifungal activities to varying extents against the four fungi and most of compounds **1**–**10** showed some antifungal activities against *R. solani*. Especially compounds **7** (Ar = 2-chloro-3-pyridyl) and **10** (Ar = 2-difluoromethyl-4-methyl-pyrazolyl) had good antifungal activity against *R. solani* and their inhibition rates were 83.58 and 88.81%, respectively. In addition, compound **10** displayed a much higher antifungal activity against *R. solani* (88.81%) than against *F. oxysporum*, *A. tenuissima*, and *A. solani* (50.60, 43.27, and 47.21%). To further analyze the antifungal activities of the compounds, compounds **7** and **10** that exhibited stronger antifungal activities against *R. solani* were evaluated by determination of EC_50_ values. The corresponding EC_50_ values are listed in Table 2. Compound **10** (EC_50_ = 10.34 mg/L) had better antifungal activity against *R. solani* than compound **7** (EC_50_ = 20.37 mg/L). The results showed that compound **10** containing a 3-difluoromethyl-1-methyl-4-pyrazolyl moiety had the best antifungal activity as compared with other compounds **1**–**9** containing a different aromatic heterocyclic moiety. It was seen that compound **10** could be the novel potential lead compound against *R. solani*.

Thus, in order to further study antifungal activities of the novel benodanil-heterocyclic carboxamide hybrids, on the basis of compound **10** as the lead compound by bioisosteric modification, some substituted groups were introduced, such as fluorine atom, bromine atom, and so on, to replace the 3-chloro atom in benzene ring. Antifungal activities of the novel target compounds **11**–**20** were evaluated by the growth rate method [21]. Preliminary in vitro antifungal activities of the novel target compounds **11**–**20** against *R. solani* are summarized in Table 3. In compounds **11**–**20**, compounds **17** (R = 3-CH(CH_3_)_2_) and **18** (R = 3-CF_3_) displayed promising antifungal activities (inhibition rate ≥ 80%) against *R. solani* at a dosage of 50 mg/L, and the inhibition rate of compound **17** (90.30%) was found to be higher than that of compound **10** (88.81%). Their EC_50_ values also displayed that compounds **17** (EC_50_ = 6.32 mg/L) and **18** (EC_50_ = 6.06 mg/L) had better antifungal activities against *R. solani* than the lead compound **10** (R = 3-Cl, EC_50_ = 10.34 mg/L) and the commercial SDHI benodanil (EC_50_ = 6.38 mg/L), as shown in Table 2.

### 2.3. Effects on the Mycelium Morphology

**Figure 3:**
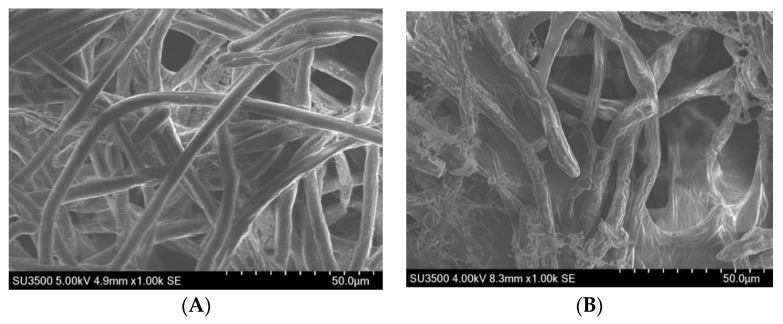
Scanning electron microscopes (SEM). (**A**) Negative control; (**B**) Treated colony with compound **17** at 10 mg/L.

Scanning electron microscope (SEM) images (Figure 3) showed that *R. solani* cultivated on media, without the addition of any compound, featured dense, sturdy, and smooth mycelia with a fine morphology. In contrast, the morphology of the mycelia of *R. solani* changed when it was cultured on media with the addition of compound **17** (10 mg/L) and the mycelia grew abnormally with a comparatively tenuous, wizened, and overlapping colony.

### 2.4. Fungal SDH Inhibition Activities

Furthermore, because the lead fungicide benodanil was a succinate dehydrogenase inhibitor, in order to investigate whether the novel benodanil-heterocyclic carboxamide hybrids had inhibitory activity against succinate dehydrogenase (SDH) or not, inhibition of the fungal SDH was performed. Compounds **17** and **18** were selected and tested against SDH, in vitro, from mitochondria of *R. solani*. As demonstrated in Table 4, the selected compounds **17** (IC_50_ = 52.58 mg/L) and **18** (IC_50_ = 56.86 mg/L) showed better inhibition abilities against SDH than benodanil (IC_50_ = 62.02 mg/L), proving that the SDH probably was a potent target of benodanil-heterocyclic carboxamide hybrids.

### 2.5. Molecular Docking

**Figure 4:**
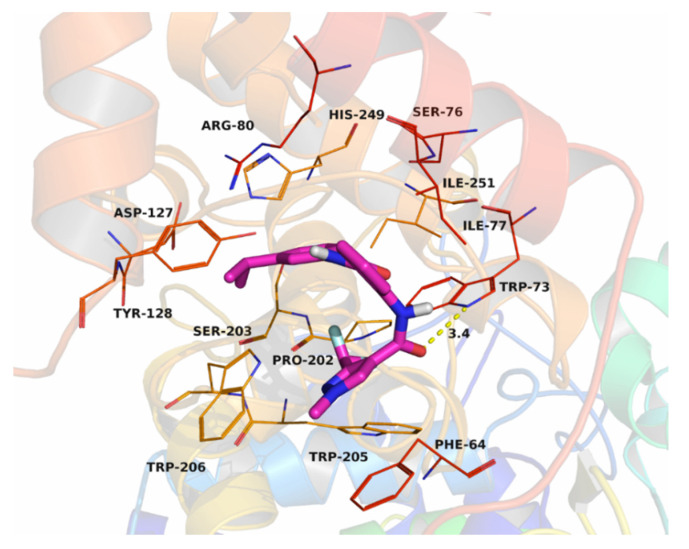
The theoretical binding mode between compound **17** and SDH by PyMoL 1.7.6.

To further survey whether the SDH is a potential target enzyme of benodanil-heterocyclic carboxamide hybrids or not, compound **17** was docked into the binding site of the SDH. The theoretical binding mode between **17** and SDH is shown in Figure 4. Compound **17** fit into the gap composed of subunit B, C, and D of SDH. The phenyl group in the middle of compound **17** occupied the hydrophobic pocket, which was composed of the residues B/Pro-202, B/Ile-251, C/Ile-77, and C/Trp-73, while the 3-(difluoromethyl)-1-methyl-1H-pyrazole scaffold of compound **17** located at another hydrophobic pocket, surrounded by the residues B/Trp-205, B/Trp-206, C/Phe-64, and C/Trp-73, formed a stable hydrophobic binding. A detailed analysis showed that a π–π stacking interaction was observed between the pyrazole group in compound **17** and the sidechain of the residue C/Phe-64. In addition, the isopropylphenyl group in compound **17** formed a cation–π interaction with C/Arg-80. Importantly, one hydrogen bond interaction was observed between compound **17** and the residue C/Trp-73 (bond length 3.4 Å), which was the main interaction between them (Figure 4). All these interactions helped compound **17** to anchor in the binding site of SDH.

## 3. Materials and Methods

### 3.1. Reagents and Instruments

Some ingredients were purchased from commercial sources unless otherwise specified. All solvents and liquid reagents were dried by standard methods in advance and distilled before use. All fungi were obtained from the Institute of Pesticide and Crop Protection, Sichuan University. ^1^H NMR spectra were recorded in DMSO-*d*_6_ solution on a Bruker 400 MHz (Bruker Corporation, Billerica, MA, USA), using tetramethylsilane (TMS) as an internal standard, and chemical shift values (δ) were given in parts per million (ppm). The following abbreviations were used to designate chemical shift multiplicities: s = singlet, d = doublet, t = triplet, q = quartet, and m = multiple. Thin-layer chromatography (TLC) was performed on silica gel 60 F254 (Qingdao Marine Chemical Ltd., Qingdao, China). Column chromatography (CC) purification was performed over silica gel (200–300 mesh, Qingdao Marine Chemical Ltd.). MS data were obtained using a MALDI-TOF/TOF mass spectrometer (Shimadzu Corporation, Kyoto, Japan). Scanning electron microscopy was performed using a SU3500 Hitachi (Hitachi Group, Tokyo, Japan)

### 3.2. General Synthetic Procedure for Key Intermediates

#### 3.2.1. Synthesis of the Intermediate **c**

Compound **a** (20 mmol) was dissolved in dichloromethane (20 mL) at room temperature, then, *N*,*N*-dimethylformamide (5–6 drops) and thionyl chloride (15 mL) were added, in that order, and the resulting mixture was stirred at 70 °C, for 3 h. The remaining solvents dichloromethane and thionyl chloride were removed by distillation under reduced pressure to yield the corresponding compound **b**.

To a solution of 3-chloroaniline (0.02 mol) in triethylamine (5 mL) and dichloromethane (15 mL) in an ice bath, freshly prepared compound **b** was added dropwise, and the resulting mixture was stirred at room temperature for 3 h. Then, the mixture was filtered, and successively washed with aqueous sodium hydroxide (5%, 3 × 15 mL) and water (2 × 15 mL). The organic phase was, then, dried over anhydrous sodium sulfate, filtered, and evaporated in vacuo to remove dichloromethane. Finally, the residue was purified by column chromatography to yield the intermediate **c**.

#### 3.2.2. Synthesis of Intermediate **d**

Compound **c** (15 mmol), reductive iron powder (15 mmol), NH_4_Cl (45 mmol), and ethanol aqueous solution (75%, 60 mL) were added in a flask. The reaction proceeded with refluxing for 3 h, at 90 °C. When the reaction finished, the mixtures were cooled to room temperature and extracted with CH_2_Cl_2_ (3 × 20 mL) and the organic phase was evaporated in a vacuum to obtain compound **d**.

### 3.3. General Synthetic Procedures for Target Compounds **1**–**20**

Intermediate **d** (10 mmol), EDCI (10 mmol), DMAP (0.1 mmol), and aromatic acid (10 mmol) were dissolved in DCM (CH_2_Cl_2_, 30 mL). The mixture was stirred at room temperature for 6 h. The target compounds **1**–**10** were purified via column chromatography. Similarly, the intermediate **b**, **e**, **f**, and target compounds **11**–**20** were synthesized by using the same methods. The spectrogram of target compounds **1**–**20** are presented as Supporting Information.

Compound **1**: Yield, 83.6%; white solid; ^1^H NMR (400 MHz, DMSO) δ 11.52 (s, 1H), 10.71 (s, 1H), 8.49–8.45 (m, 1H), 8.02 (dd, *J* = 1.7, 0.7 Hz, 1H), 7.92 (dd, *J* = 7.9, 1.4 Hz, 1H), 7.90 (t, *J* = 2.1 Hz, 1H), 7.70–7.66 (m, 1H), 7.66–7.61 (m, 1H), 7.43 (t, *J* = 8.1 Hz, 1H), 7.32 (dd, *J* = 7.6, 1.0 Hz, 1H), 7.29–7.28 (m, 1H), 7.22 (ddd, *J* = 8.0, 2.1, 0.9 Hz, 1H), 6.74 (dd, *J* = 3.5, 1.8 Hz, 1H); ^13^C NMR (100 MHz, DMSO-*d*_6_) δ 167.88, 156.10, 147.77, 146.59, 140.44, 138.41, 133.37, 132.92, 130.82, 129.50, 124.38, 123.79, 122.83, 121.64, 120.84, 119.80, 115.90, 113.08; ESI-HRMS: *m/z* [M + Na]^+^ calcd. for [C_18_H_13_ClN_2_NaO_3_]: 363.0512; found: 363.0480.

Compound **2**: Yield, 75.5%; white solid; ^1^H NMR (400 MHz, DMSO) δ 11.01 (s, 1H), 10.64 (s, 1H), 8.35 (s, 1H), 8.22 (d, *J* = 8.1 Hz, 1H), 7.90 (s, 1H), 7.86 (d, *J* = 8.6 Hz, 1H), 7.85–7.80 (m, 1H), 7.67 (d, *J* = 8.2 Hz, 1H), 7.61 (dd, *J* = 11.4, 4.2 Hz, 1H), 7.40 (t, *J* = 8.1 Hz, 1H), 7.30 (t, *J* = 7.3 Hz, 1H), 7.19 (dd, *J* = 8.0, 1.2 Hz, 1H), 6.86 (d, *J* = 1.0 Hz, 1H); ^13^C NMR (100 MHz, DMSO-*d*_6_) δ 167.73, 160.58, 146.39, 145.23, 140.63, 138.14, 133.35, 132.58, 130.78, 129.37, 124.53, 124.18, 124.06, 123.42, 122.57, 120.62, 119.58, 108.98; ESI-HRMS: *m/z* [M + Na]^+^ calcd. for [C_18_H_13_ClN_2_NaO_3_]: 363.0512; found: 363.0481.

Compound **3**: Yield, 69.8%; grey solid; ^1^H NMR (400 MHz, DMSO) δ 11.03 (s, 1H), 10.65 (s, 1H), 8.36 (d, *J* = 7.6 Hz, 1H), 7.89 (t, *J* = 1.9 Hz, 1H), 7.87 (d, *J* = 1.2 Hz, 1H), 7.73–7.66 (m, 1H), 7.65 (d, *J* = 2.0 Hz, 1H), 7.60 (s, 1H), 7.41 (t, *J* = 8.1 Hz, 1H), 7.29 (dd, *J* = 7.6, 0.9 Hz, 1H), 7.24–7.17 (m, 1H), 6.77 (d, *J* = 2.0 Hz, 1H), 2.56 (s, 3H); ^13^C NMR (100 MHz, DMSO-*d*_6_) δ 167.96, 161.71, 157.45, 141.93, 140.52, 138.76, 133.34, 132.72, 130.80, 129.37, 124.28, 123.63, 123.36, 121.92, 120.74, 119.69, 116.45, 109.04, 13.66; ESI-HRMS: *m/z* [M + Na]^+^ calcd. for [C_19_H_15_ClN_2_NaO_3_]: 377.0669; found: 377.0633.

Compound **4**: Yield, 77.9%; yellow solid; ^1^H NMR (400 MHz, DMSO) δ 11.42 (s, 1H), 10.67 (s, 1H), 8.27 (dd, *J* = 8.2, 0.7 Hz, 1H), 7.91 (d, *J* = 1.2 Hz, 1H), 7.90–7.89 (m, 1H), 7.88 (d, *J* = 1.3 Hz, 1H), 7.79 (dd, *J* = 3.8, 1.1 Hz, 1H), 7.71–7.65 (m, 1H), 7.65–7.55 (m, 1H), 7.41 (t, *J* = 8.1 Hz, 1H), 7.31 (td, *J* = 7.7, 1.1 Hz, 1H), 7.26 (dd, *J* = 5.0, 3.8 Hz, 1H), 7.20 (ddd, *J* = 8.0, 2.1, 0.9 Hz, 1H); ^13^C NMR (100 MHz, DMSO-*d*_6_) δ 167.84, 159.92, 140.52, 139.86, 139.75, 138.27, 133.35, 132.73, 132.68, 130.79, 129.43 129.35, 128.86, 124.29, 124.08, 122.35, 120.76, 119.71; ESI-HRMS: *m/z* [M + Na]^+^ calcd. for [C_18_H_13_ClN_2_NaO_2_S]: 379.0284; found: 379.0335.

Compound **5**: Yield, 68.7%; white solid; ^1^H NMR (400 MHz, DMSO) δ 11.26 (s, 1H), 10.66 (s, 1H), 8.32 (d, *J* = 8.1 Hz, 1H), 8.26 (dd, *J* = 2.9, 1.3 Hz, 1H), 7.89 (t, *J* = 1.8 Hz, 1H), 7.87 (d, *J* = 1.3 Hz, 1H), 7.71 (dd, *J* = 5.1, 2.9 Hz, 1H), 7.67 (dd, *J* = 8.2, 1.0 Hz, 1H), 7.64–7.59 (m, 1H), 7.52 (dd, *J* = 5.1, 1.3 Hz, 1H), 7.40 (t, *J* = 8.1 Hz, 1H), 7.30 (td, *J* = 7.7, 1.1 Hz, 1H), 7.20 (ddd, *J* = 8.0, 2.1, 0.9 Hz, 1H); ^13^C NMR (100 MHz, DMSO-*d*_6_) δ 167.89, 160.85, 140.56, 138.56, 138.00, 133.34, 132.70, 130.79, 130.45, 129.43, 128.33, 126.65, 124.25, 123.97, 123.90, 122.26, 120.73, 119.70; ESI-HRMS: *m/z* [M + Na]^+^ calcd. for [C_18_H_13_ClN_2_NaO_2_S]: 379.0284; found: 379.0369.

Compound **6**: Yield, 70.2%; white solid; ^1^H NMR (400 MHz, DMSO) δ 11.37 (s, 1H), 10.67 (s, 1H), 9.09 (d, *J* = 2.0 Hz, 1H), 8.78 (dd, *J* = 4.8, 1.6 Hz, 1H), 8.24 (ddd, *J* = 15.6, 8.9, 4.4 Hz, 2H), 7.89 (d, *J* = 1.7 Hz, 1H), 7.86 (d, *J* = 7.8 Hz, 1H), 7.66–7.58 (m, 3H), 7.39 (d, *J* = 8.1 Hz, 1H), 7.36–7.31 (m, 1H), 7.22–7.13 (m, 1H); ^13^C NMR (100 MHz, DMSO-*d*_6_) δ 168.82, 160.22, 149.78, 146.29, 143.85, 142.01, 140.79, 138.80, 136.32, 135.45, 134.14, 131.43, 129.80, 126.64, 124.19, 122.41, 121.19, 119.81, 118.34; ESI-HRMS: *m/z* [M + Na]^+^ calcd. for [C_19_H_14_ClN_3_NaO_2_]: 374.0672; found: 374.0638.

Compound **7**: Yield, 82%; orange solid; ^1^H NMR (400 MHz, DMSO) δ 10.92 (s, 1H), 10.63 (s, 1H), 8.68–8.48 (m, 1H), 8.25 (dt, *J* = 7.7, 1.9 Hz, 1H), 8.09–8.00 (m, 1H), 8.00–7.86 (m, 1H), 7.77 (d, *J* = 7.6 Hz, 1H), 7.59 (ddd, *J* = 10.2, 7.7, 5.0 Hz, 2H), 7.36 (td, *J* = 8.0, 1.7 Hz, 1H), 7.24–7.05 (m, 1H), 6.82–6.54 (m, 1H), 6.37 (s, 1H); ^13^C NMR (100 MHz, DMSO-*d*_6_) δ 167.70, 160.13, 145.34, 141.66, 137.58, 135.72, 134.36, 133.03, 132.51, 129.31, 125.45, 124.35, 123.52, 123.25, 119.10, 116.49, 114.37, 112.29, 110.09; ESI-HRMS: *m/z* [M + Na]^+^ calcd. for [C_19_H_13_Cl_2_N_3_NaO_2_]: 408.0283; found: 408.0257.

Compound **8**: Yield, 78.5%; grey solid; ^1^H NMR (400 MHz, DMSO) δ 10.90 (d, *J* = 8.8 Hz, 1H), 10.63 (d, *J* = 3.6 Hz, 1H), 8.53 (d, *J* = 4.7 Hz, 1H), 8.12–7.97 (m, 2H), 7.90 (d, *J* = 1.9 Hz, 1H), 7.76 (dd, *J* = 5.1, 2.5 Hz, 1H), 7.59 (ddd, *J* = 12.4, 9.6, 6.2 Hz, 3H), 7.36 (t, *J* = 8.0 Hz, 2H), 7.15 (dd, *J* = 7.7, 1.7 Hz, 1H); ^13^C NMR (100 MHz, DMSO-*d*_6_) δ 168.35, 149.95, 147.05, 143.83, 141.21, 135.22, 133.26, 132.83, 130.63, 129.18, 123.44, 121.82, 120.19, 119.14, 117.11, 115.54, 112.02, 101.88; ESI-HRMS: *m/z* [M + Na]^+^ calcd. for [C_19_H_13_BrClN_3_NaO_2_]: 451.9777; found: 452.0810.

Compound **9**: Yield, 81.9%; white solid; ^1^H NMR (400 MHz, DMSO) δ 11.15 (s, 1H), 10.65 (s, 1H), 7.94 (dd, *J* = 16.2, 8.2 Hz, 2H), 7.85–7.75 (m, 1H), 7.62 (d, *J* = 8.1 Hz, 2H), 7.40–7.34 (m, 2H), 7.20–7.12 (m, 1H), 2.75 (s, 3H); ^13^C NMR (100 MHz, DMSO-*d*_6_) δ 168.69, 167.04, 141.16, 140.77, 136.31, 133.31, 132.25, 130.75, 130.64, 129.36, 129.17, 125.48, 124.02, 123.48, 120.33, 119.27, 119.16, 117.23, 31.11; ESI-HRMS: *m/z* [M + Na]^+^ calcd. for [C_19_H_13_ClFN_3_NaO_2_S]: 462.0267; found: 462.0248.

Compound **10**: Yield, 67%; white solid; ^1^H NMR (400 MHz, DMSO) δ 10.94 (s, 1H), 10.65 (s, 1H), 8.39 (s, 1H), 8.19 (d, *J* = 8.2 Hz, 1H), 7.91 (s, 1H), 7.86 (d, *J* = 7.7 Hz, 1H), 7.65 (d, *J* = 8.2 Hz, 1H), 7.63–7.55 (m, 1H), 7.40 (t, *J* = 8.1 Hz, 1H), 7.30 (dd, *J* = 6.1, 1.4 Hz, 1H), 7.25–7.20 (m, 1H), 7.20–7.14 (m, 1H), 3.99 (s, 3H); ^13^C NMR (100 MHz, DMSO-*d*_6_) δ 167.66, 160.05, 138.05, 136.58, 132.85, 132.49, 130.72, 130.62, 129.33, 124.56, 124.07, 122.72, 116.97, 116.95, 116.61, 110.06, 108.13, 107.87, 31.11; ESI-HRMS: *m/z* [M + Na]^+^ calcd. for [C_19_H_15_ClF_2_N_4_NaO_2_]: 427.0749; found: 427.0728.

Compound **11**: Yield, 68.6%; white solid; ^1^H NMR (400 MHz, DMSO) δ 10.95 (s, 1H), 10.67 (s, 1H), 8.71 (s, 1H), 8.37 (s, 1H), 8.22–8.16 (m, 1H), 7.85 (dd, *J* = 7.8, 1.3 Hz, 1H), 7.70 (dt, *J* = 11.7, 2.2 Hz, 1H), 7.63–7.55 (m, 1H), 7.50 (dd, *J* = 8.2, 0.9 Hz, 1H), 7.40 (ddd, *J* = 16.3, 7.5, 1.9 Hz, 1H), 7.35–7.27 (m, 1H), 7.01–6.91 (m, 1H), 3.98 (s, 3H); ^13^C NMR (100 MHz, DMSO-*d*_6_) δ 167.72, 160.10, 157.02, 139.96, 137.78, 136.67, 132.97, 132.44, 130.31, 129.34, 125.80, 125.18, 124.80, 124.24, 123.04, 120.75, 117.33, 110.03, 31.11; ESI-HRMS: *m/z* [M + Na]^+^ calcd. for [C_19_H_15_F_3_N_4_NaO_2_]: 411.1045; found: 411.1022.

Compound **12**: Yield, 70.5%; white solid; ^1^H NMR (400 MHz, DMSO) δ 10.89 (s, 1H), 10.62 (s, 1H), 8.71 (s, 1H), 8.45–8.30 (m, 1H), 8.16 (d, *J* = 6.9 Hz, 1H), 8.02 (s, 1H), 7.84 (d, *J* = 7.8 Hz, 1H), 7.68 (d, *J* = 4.0 Hz, 1H), 7.64–7.55 (m, 1H), 7.35–7.31 (m, 1H), 7.29 (d, *J* = 7.6 Hz, 1H), 7.24–7.08 (m, 1H), 3.97 (s, 3H); ^13^C NMR (100 MHz, DMSO-*d*_6_) δ 167.62, 163.36, 160.06, 140.75, 137.98, 136.58, 132.90, 132.48, 131.05, 129.37, 127.10, 124.71, 124.11, 123.56, 121.80, 120.04,112.58, 109.12, 30.14; ESI-HRMS: *m/z* [M + Na]^+^ calcd. for [C_19_H_15_BrF_2_N_4_NaO_2_]: 471.0244; found: 471.0211.

Compound **13**: Yield, 77.7%; white solid; ^1^H NMR (400 MHz, DMSO) δ 11.16 (s, 1H), 10.56 (s, 1H), 8.71 (s, 1H), 8.34 (s, 1H), 8.27 (d, *J* = 7.4 Hz, 1H), 7.89 (d, *J* = 6.8 Hz, 1H), 7.73 (s, 2H), 7.58 (d, *J* = 6.5 Hz, 1H), 7.29 (s, 1H), 7.26–7.02 (m, 2H), 3.98 (s, 3H); ^13^C NMR (100 MHz, DMSO-*d*_6_) δ 167.50, 159.99, 145.28, 138.39, 136.58, 135.29, 135.26, 132.73, 132.50, 129.27, 123.89, 123.77, 123.50, 123.42, 122.32, 116.68, 115.79, 115.576, 31.11; ESI-HRMS: *m/z* [M + Na]^+^ calcd. for [C_19_H_15_F_3_N_4_NaO_2_]: 411.1045; found: 411.1013.

Compound **14**: Yield, 79%; yellow solid; ^1^H NMR (400 MHz, DMSO) δ 11.03 (d, *J* = 10.4 Hz, 1H), 10.62 (s, 1H), 8.35 (s, 1H), 8.26–8.18 (m, 1H), 7.86 (d, *J* = 7.8 Hz, 1H), 7.76 (d, *J* = 8.8 Hz, 2H), 7.63–7.54 (m, 1H), 7.47–7.43 (m, 1H), 7.43–7.38 (m, 1H), 7.24 (dt, *J* = 50.7, 5.8 Hz, 2H), 3.97 (s, 3H); ^13^C NMR (100 MHz, DMSO-*d*_6_) δ 167.56, 160.01, 156.02, 145.88, 138.19, 138.03, 132.79, 132.49, 129.33, 128.97, 128.23, 124.25, 123.99, 122.93, 122.56, 116.81, 110.05, 108.34, 31.12; ESI-HRMS: *m/z* [M + Na]^+^ calcd. for [C_19_H_15_ClF_2_N_4_NaO_2_]: 427.0749; found: 427.0729.

Compound **15**: Yield, 59.7%; white solid; ^1^H NMR (400 MHz, DMSO) δ 11.02 (s, 1H), 10.62 (s, 1H), 8.72 (s, 1H), 8.35 (s, 1H), 8.22 (d, *J* = 7.9 Hz, 1H), 7.92–7.81 (m, 1H), 7.71 (d, *J* = 8.9 Hz, 2H), 7.58 (ddd, *J* = 8.9, 7.7, 1.6 Hz, 2H), 7.32–7.26 (m, 1H), 7.37–7.04 (m, 1H), 3.98 (s, 3H); ^13^C NMR (100 MHz, DMSO-*d*_6_) δ 167.57, 163.36, 160.01, 138.45, 138.18, 136.58, 132.79, 132.49, 131.88, 129.33, 124.26, 123.99, 123.29, 122.56, 116.65, 116.35, 112.33, 110.00, 31.10; ESI-HRMS: *m/z* [M + Na]^+^ calcd. for [C_19_H_15_BrF_2_N_4_NaO_2_]: 471.0244; found: 471.0210.

Compound **16**: Yield, 80.4%; white solid; ^1^H NMR (400 MHz, DMSO) δ 11.16 (s, 1H), 10.44 (s, 1H), 8.35 (s, 1H), 8.26 (d, *J* = 7.5 Hz, 1H), 7.90–7.84 (m, 1H), 7.61–7.55 (m, 1H), 7.49 (dd, *J* = 23.8, 15.8 Hz, 2H), 7.32–7.11 (m, 3H), 6.96 (d, *J* = 7.5 Hz, 1H), 3.97 (s, 3H), 2.31 (s, 3H); ^13^C NMR (100 MHz, DMSO-*d*_6_) δ 167.55, 160.00,148.92, 145.28, 138.81, 138.37, 138.27, 132.74, 132.43, 129.32, 128.91, 125.39, 123.95, 123.89, 122.31, 122.11, 118.80, 110.07, 31.12, 21.59; ESI-HRMS: *m/z* [M + Na]^+^ calcd. for [C_20_H_18_F_2_N_4_NaO_2_]: 407.1296; found: 407.1268.

Compound **17**: Yield, 79.4%; white solid; ^1^H NMR (400 MHz, DMSO) δ 11.17 (s, 1H), 10.44 (s, 1H), 8.35 (s, 1H), 8.26 (d, *J* = 8.3 Hz, 1H), 7.89 (d, *J* = 7.8 Hz, 1H), 7.63–7.54 (m, 1H), 7.52 (s, 1H), 7.43–7.16 (m, 1H), 7.32–7.27 (m, 1H), 7.03 (d, *J* = 7.7 Hz, 1H), 3.97 (s, 3H), 2.93–2.80 (m, 1H), 1.21 (d, *J* = 6.9 Hz, 6H); ^13^C NMR (100 MHz, DMSO-*d*_6_) δ 167.51, 160.00, 149.34, 144.94, 138.85, 138.33, 132.78, 132.40, 129.32, 128.96, 124.04, 123.89, 122.76, 122.34, 119.59, 119.27, 116.71, 110.52, 40.12, 33.88, 24.27; ^19^F NMR (225 MHz, DMSO-*d*_6_) δ −114.10 (d, *J* = 31.5Hz, 2F); ESI-HRMS: *m/z* [M + Na]^+^ calcd. for [C_22_H_22_F_2_N_4_NaO_2_]: 435.1609; found: 435.1590.

Compound **18**: Yield, 66.9%; white solid; ^1^H NMR (400 MHz, DMSO) δ 10.84 (s, 1H), 10.76 (s, 1H), 8.38 (s, 1H), 8.12 (d, *J* = 6.1 Hz, 2H), 7.99 (d, *J* = 8.2 Hz, 1H), 7.86 (d, *J* = 7.8 Hz, 1H), 7.66–7.55 (m, 2H), 7.48 (d, *J* = 7.8 Hz, 1H), 7.40–7.13 (m, 1H), 7.34–7.29 (m, 1H), 3.97 (s, 3H); ^13^C NMR (100 MHz, DMSO-*d*_6_) δ 167.59, 163.90, 152.82, 148.67, 140.72, 137.85, 135.59, 133.33, 132.48, 130.78, 130.20, 129.46, 125.58, 124.58, 124.32, 124.10, 123.00, 120.50, 119.46, 31.12; ^19^F NMR (225 MHz, DMSO-*d*_6_) δ -114.10 (d, *J* = 33.8Hz, 2F), −61.27 (s, 3F); ESI-HRMS: *m/z* [M + Na]^+^ calcd. for [C_20_H_15_F_5_N_4_NaO_2_]: 461.1013; found: 461.0971.

Compound **19**: Yield, 73.4%; white solid; ^1^H NMR (400 MHz, DMSO) δ 10.84 (s, 1H), 10.74 (s, 1H), 8.37 (d, *J* = 20.5 Hz, 1H), 8.10 (dd, *J* = 12.0, 5.1 Hz, 1H), 7.84 (d, *J* = 7.6 Hz, 1H), 7.68 (d, *J* = 2.2 Hz, 1H), 7.62 (dd, *J* = 9.7, 5.6 Hz, 2H), 7.42–7.11 (m, 2H), 5.77 (d, *J* = 1.6 Hz, 1H), 3.98 (s, 3H); ^13^C NMR (100 MHz, DMSO-*d*_6_) δ 167.62, 163.35, 160.09, 139.36, 137.81, 136.58, 132.96, 132.49, 131.27, 130.95, 129.35, 125.91, 125.01, 124.20, 123.02, 122.35, 121.15, 110.04, 31.11; ESI-HRMS: *m/z* [M + Na]^+^ calcd. for [C_19_H_14_Cl_2_F_2_N_4_NaO_2_]: 461.0360; found: 461.0328.

Compound **20**: Yield, 77.2%; dark grey solid; ^1^H NMR (400 MHz, DMSO) δ 10.73 (s, 2H), 8.41 (s, 1H), 8.09 (s, 1H), 7.81 (s, 2H), 7.59 (d, *J* = 6.7 Hz, 1H), 7.49–7.04 (m, 3H), 5.77 (s, 1H), 3.98 (s, 3H); ^13^C NMR (100 MHz, DMSO-*d*_6_) δ 167.04, 164.62, 152.65, 151.28, 148.20, 146.89, 140.94, 140.68, 138.69, 133.30, 133.01, 130.72, 130.66, 129.32, 127.07, 123.67, 120.16, 119.09, 32.26; ESI-HRMS: *m/z* [M + Na]^+^ calcd. for [C_19_H_14_Cl_2_F_2_N_4_NaO_2_]: 461.0360; found: 461.0334.

### 3.4. Antifungal Bioassay

All synthesized compounds **1**–**20** were screened for their in vitro antifungal activities against four fungi, including *R. solani*, *F. oxysporum*, *A. tenuissima*, and *A. solani*. by the mycelium growth rate method. All screened compounds were dissolved in acetone (1 mL), and the solutions were diluted with aqueous 1% Tween 60, and then were added to sterile potato dextrose agar (PDA, 49 mL). For primary screening, the compounds were tested at a concentration of 50 mg/L. All fungi species were cultivated in PDA at 28 ± 1 °C, for 2 days, to produce new mycelium before the antifungal assays were conducted. Next, mycelia dishes of about 8 mm diameter were cut from the culture medium and were picked up with a sterilized inoculating needle and inoculated in the center of the PDA plates. The inoculated plates were incubated at 28 ± 1 °C, for 24 h. Acetone in sterile aqueous 1% Tween 60 served as the negative control, whereas thifluzamide served as the positive control. Each sample was screened in three replicates and each colony diameter of the three replicates was measured four times by the cross-bracketing method. The data was statistically analyzed and the corrected inhibitory rates (I) were calculated using the following formula: I (%) = [(C-T)/(C-8 mm)] × 100, where C represents the average diameter of mycelia in the blank test, T represents the average diameter of mycelia on treated PDA media. The results of the antifungal tests are summarized in Table 1 and Table 3, and the EC_50_ values of some compounds were calculated and are listed in Table 2.

### 3.5. Scanning Electron Microscopy

The effect of compound 1**7** on mycelial morphology was observed, according to the previously reported method [22]. Five 0.7 cm cakes of *R. solani* were put into PDB liquid medium and cultured at 28 °C, 160 r/min, for 3 days. Compound **17** dissolved in DMSO was added into 50 mL PDB liquid culture medium, the final concentrations of the agent were 10 mg/L, and the control was DMSO of the same volume, and the culture continued for 24 h. The mycelium was taken out, 2.5% glutaraldehyde was fixed at 4 °C, for 4–6 h, the mycelium was washed with PBS buffer for 2–3 times, then dehydrated with ethanol gradient (30%, 50%, 70%, 85%, and 90% once respectively, 100% twice, 20 min each time); the dehydrated mycelium was dried in vacuum, finally gold plating was carried out. The mycelia were observed and photographed on a SU3500 Hitachi scanning electron microscope.

### 3.6. Assay of Enzyme Inhibitory

#### 3.6.1. Isolation of *R. solani*

Fungus mitochondria were isolated, according to a previously reported method. Cultures were inoculated at 0.05 OD600 nm and grown on a reciprocal shaker (180 rpm, 25 °C) for 5 days in Sabouraud maltose broth (SMB) medium. Cells were harvested by vacuum filtration and disrupted in liquid nitrogen using a mortar and pestle. The resultant powder was resuspended to10 % *w/v* in mitochondrial extraction buffer (10 mM KH_2_PO_4_, pH 7.2, 10 mM KCl, 10 mM MgCl_2_, 0.5 M sucrose, 0.2 mM EDTA, 2 mM PMSF). The extract was clarified by centrifugation (5000 *g*, 4 °C, for 10 min, 2 times), and then intact mitochondria were pelleted at 10,000 *g* for 20 min, at 4 °C, and resuspended in the same buffer. Mitochondrial suspensions were brought to a concentration of 10 mg/mL and stored at −80 °C until use. The activity of SDH was found to remain stable for months.

#### 3.6.2. Succinate:Ubiquinone/DCPIP Activity Inhibition

Mitochondrial suspensions were diluted 1/20 in extraction buffer and preactivated at 30 °C for 30 min in the presence of 10 mM succinate. Succinate:ubiquinone/DCPIP activity inhibition measurements were performed by adding 10 μL of preactivated mitochondria to 200 μL of assay buffer (50 mM phosphate/sodium, pH 7.2, 250 mM sucrose, 3 mM NaN_3_, 10 mM succinate) supplemented with 140 μM dichlorophenolindophenol (DCPIP) and 1 mM 2,3-dimethoxy-5-methyl-1,4-benzoquinone (Q_0_). Inhibitor concentrations ranged between 0.0050 and 15 mg/L, with uniform 5× dilution factor steps (six inhibitor concentrations + DMSO control). A total of 96 well plates were pre-equilibrated at reaction temperature (30 °C) for 10 min before the reactions were started by the addition of 10 μL of preactivated *R. solani* mitochondrial suspension. DCPIP reduction was conducted at 30 °C and monitored at 595 nm. Calculated absorbance slopes (OD/h) were used for half-inhibitory concentration (IC_50_) calculations using GraphPad Prism 5.0 software (GraphPad Software, San Diego, CA, USA).

### 3.7. Homology Modeling and Molecular Docking

#### 3.7.1. Homology Modeling

The NCBI protein database (http://www.ncbi.nlm.nih.gov/protein/) was used to search the SDH amino acid sequence of *R. solani*. The employed protein sequence was CUA72490.1, CUA71217.1, CUA73421.1, and CUA73959.1 reported by Wibberg. The BLAST server (http://blast.ncbi.nlm.nih.gov) was used to search a template for the chain. We applied SDH from avian (PDB ID:1YQ3) as the template, and the homology of amino acid sequence was aligned. Homology modeling of SDH from *R. solani* was carried out using MODELER 9.15 (http://salilab.org/modeller/).

#### 3.7.2. Molecular Docking

Molecular docking studies were performed to investigate the binding mode of compound **17** to SDH using Autodock vina 1.1.2 (Scripps Research, San Diego, CA, USA) The three-dimensional (3D) structures of compound **17** were drawn by ChemBioDraw Ultra 14.0 (PerkinElmer, Waltham, MA, USA) and ChemBio3D Ultra 14.0 softwares (PerkinElmer, Waltham, MA, USA) The AutoDock Tools 1.5.6 package (http://mgltools.scripps.edu) was employed to generate the docking input files. The search grid of SDH was identified as center_x: 86.459, center_y: 65.6, and center_z: 85.537 with dimensions size_x: 15, size_y: 15, and size_z: 15. The value of exhaustiveness was set to 20. For Vina docking, the default parameters were used if it was not mentioned. The best-scoring pose, as judged by the Vina docking score, was chosen and visually analyzed using PyMOL1.7.6 software (http://www.pymol.org/).

## 4. Conclusions

In summary, a series of novel benodanil-heterocyclic carboxamide hybrids were designed, synthesized, and screened for their antifungal activity against four phytopathogenic fungi. Compounds **17** (R = 3-CH(CH_3_)_2_, EC_50_ = 6.32 mg/L) and **18** (R = 3-CF_3_, EC_50_ = 6.06 mg/L) exhibited better antifungal activities against *R. solani* than the lead benodanil (EC_50_ = 6.38 mg/L). In addition, compounds 17 (IC_50_ = 52.58 mg/L) and 18 (IC_50_ = 56.86 mg/L) showed better inhibition abilities against SDH than benodanil (IC50 = 62.02 mg/L). In the SEM images, the marked changes on the treated media with the addition of compound **17** displayed that the mycelia grew abnormally with a comparatively tenuous, wizened, and overlapping colony. This molecular docking study provided further insights into the interactions between compound **17** and SDH. The benodanil-heterocyclic carboxamide hybrids could be a class of promising lead compounds for the development of SDHIs.

## Figures and Tables

**Table 1:** Antifungal activities of the target compounds **1**–**10** against four fungi at 50 mg/L.

No.	Ar	Inhibition Rate (%)			
		*R. solani*	*F. oxysporum*	*A. tenuissima*	*A. solani*
**1**	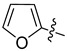	34.33 ± 0.8	28.81 ± 1.3	30.00 ± 1.1	21.55 ± 1.4
**2**	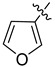	31.34 ± 0.7	33.55 ± 2.0	19.22 ± 1.7	11.06 ± 0.7
**3**	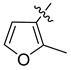	35.82 ± 1.9	27.45 ± 2.1	22.30 ± 0.9	15.47 ± 1.5
**4**	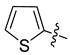	50.75 ± 2.2	12.84 ± 1.6	18.59 ± 0.8	22.53 ± 2.3
**5**	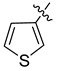	10.45 ± 0.8	20.53 ± 2.1	19.68 ± 1.1	10.80 ± 2.0
**6**	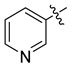	32.84 ± 1.5	11.49 ± 1.8	21.21 ± 1.5	22.201.4
**7**	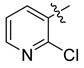	83.58 ± 1.6	47.59 ± 2.2	33.96 ± 1.4	40.78 ± 1.9
**8**	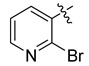	62.69 ± 1.0	39.87 ± 1.3	20.58 ± 1.5	29.96 ± 2.4
**9**	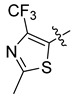	68.66 ± 2.2	42.73 ± 0.6	37.45 ± 1.4	33.44 ± 1.7
**10**	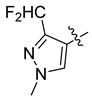	88.81 ± 0.9	50.60 ± 1.2	43.27 ± 1.9	47.21 ± 1.1

**Table 2:** EC_50_ values of some compounds with excellent effect against *R.solani.*

No.	EC_50_ (mg/L)	No.	EC_50_ (mg/L)
**7**	20.37 ± 0.4	18	6.06 ± 0.7
**10**	10.34 ± 0.7	benodanil	6.38 ± 0.6
**17**	6.32 ± 0.4		

**Table 3:** Antifungal activities of the target compounds **11**–**20** against *R. solani* at 50 mg/L.

No.	R	Inhibition Rate (%)	No.	R	Inhibition Rate (%)
**11**	3-F	40.30 ± 1.2	16	3-CH_3_	5.97 ± 0.7
**12**	3-Br	58.21 ± 1.1	17	3-CH(CH_3_)_2_	90.30 ± 1.5
**13**	4-F	62.69 ± 1.6	18	3-CF_3_	83.58 ± 1.1
**14**	4-Cl	61.19 ± 0.9	19	3,4-Cl_2_	77.61 ± 1.8
**15**	4-Br	34.33 ± 1.1	20	3–5-Cl_2_	79.10 ± 0.7

**Table 4:** IC_50_ (mg/L) values of compounds **17** and **18** against SDH of *R. solani*.

No.	IC_50_ (mg/L)
**17**	52.58 ± 1.2
**18**	56.86 ± 1.7
benodanil	62.02 ± 1.2

## Supplementary Materials

The spectrogram of title compounds **1**–**20** were presented as Supporting Information.
