# Supplementary material for: Synthesis and Biological Evaluation of Novel Benodanil-Heterocyclic Carboxamide Hybrids as a Potential Succinate Dehydrogenase Inhibitors
Source: Molecules. 2020 Sep 18;25(18):4291. doi: 10.3390/molecules25184291 (PMC7570671; doi:10.3390/molecules25184291)
Supplement: Supplementary file 1 [file molecules-25-04291-s001.pdf]

Supporting data

Synthesis and biological evaluation of novel benodanil-heterocyclic carboxamides hybrids as a potent succinate dehydrogenase inhibitor

Jian Yang, Yongtian Zhao, Jun Wan, Mingfang Jiang, Hong Jin*, Ke Tao, Taiping Hou

Key Laboratory of Bio-Resource and Eco-environment of Ministry of Education, College of Life Sciences, Sichuan University, Chengdu, 610064, China

*Corresponding Author:

E-mail address: jinhong@scu.edu.cn (H.J.).

Phone No: 86-28-85415611

1. Spectra of the target compounds 1-20

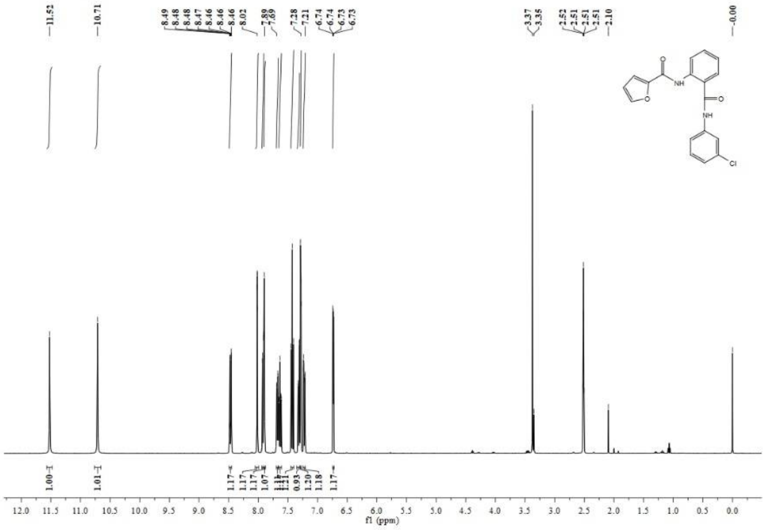

The ^{1}H NMR spectrogram of compound **1**

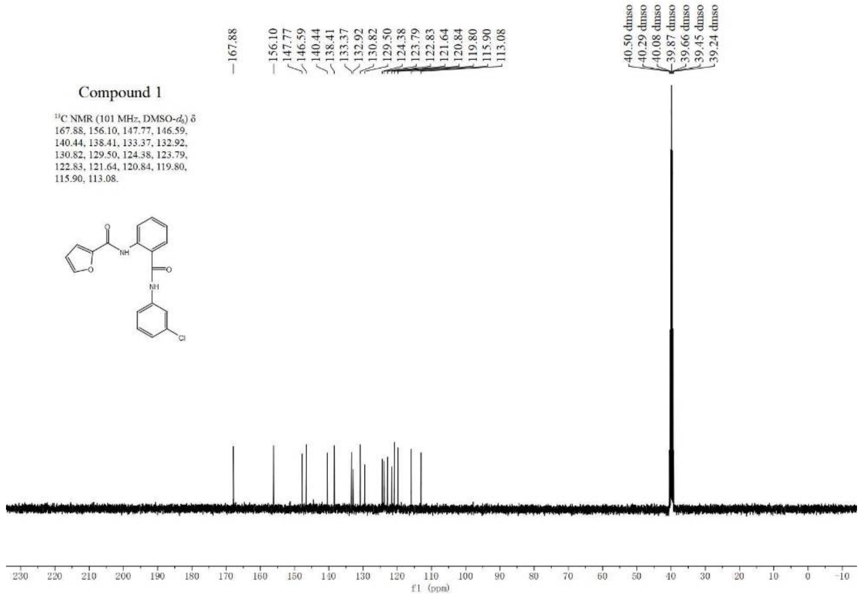

The ^{13}C NMR spectrogram of compound **1**

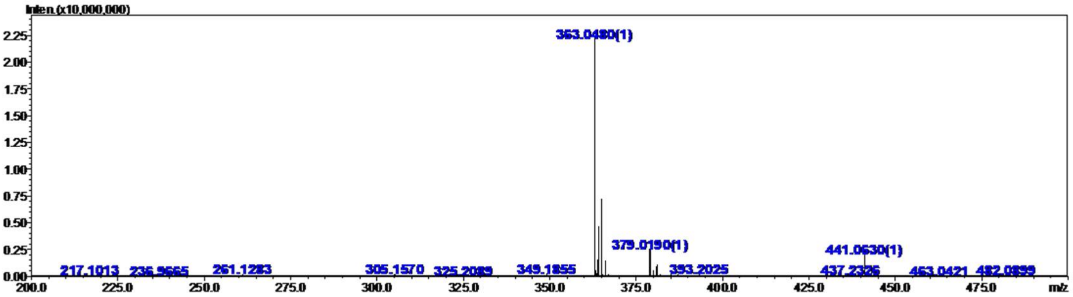

The HRMS of compound **1**

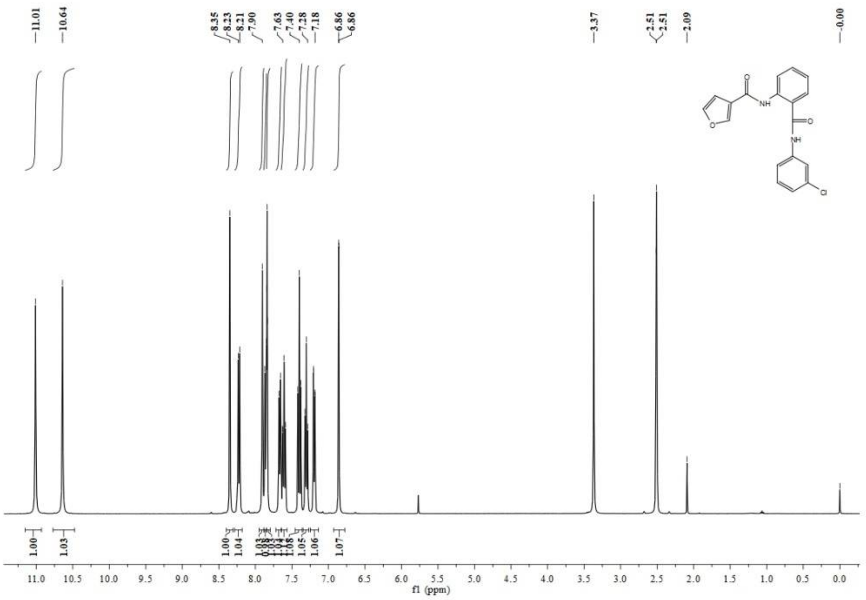

The ^{1}H NMR spectrogram of compound **2**

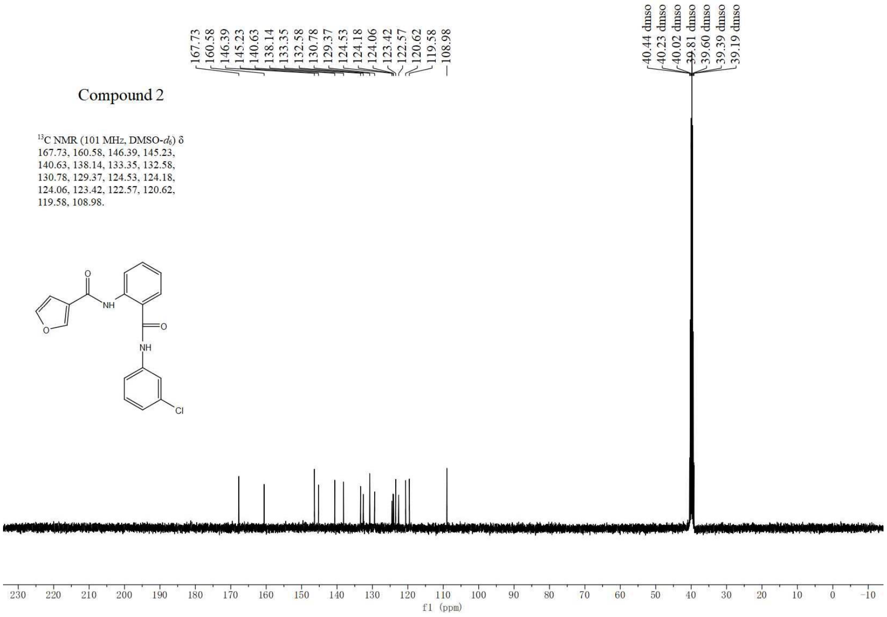

The ^{13}C NMR spectrogram of compound **2**

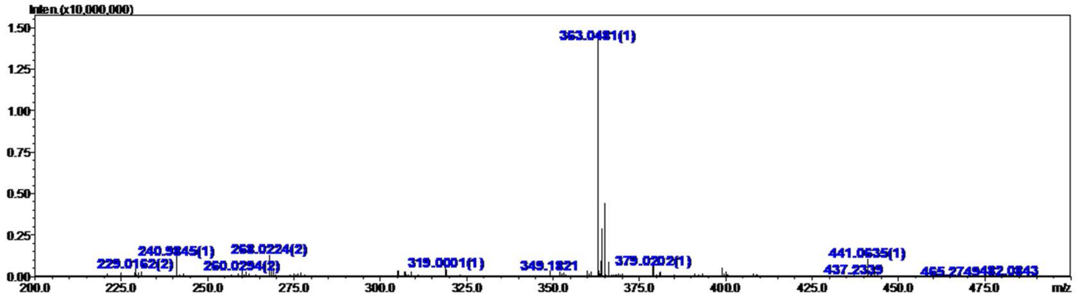

The HRMS of compound **2**

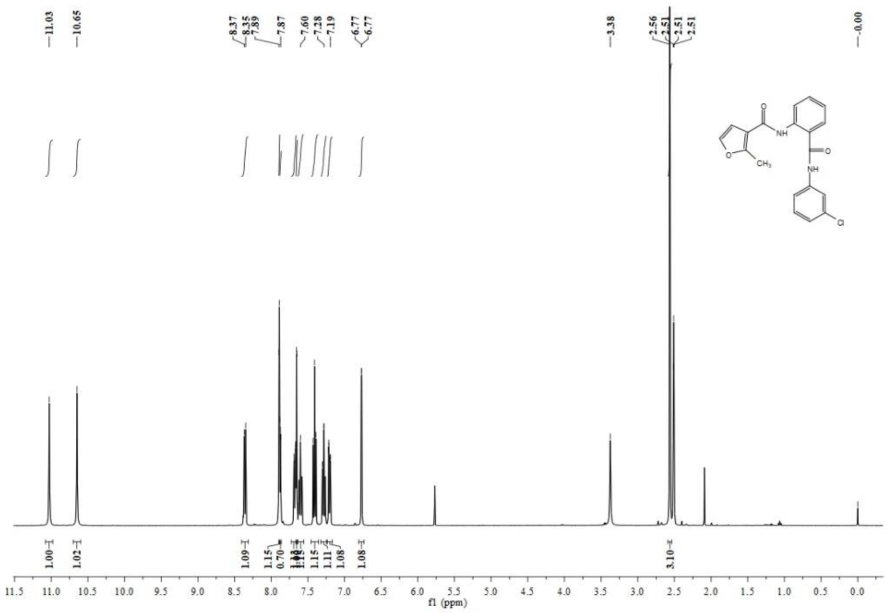

The ^{1}H NMR spectrogram of compound **3**

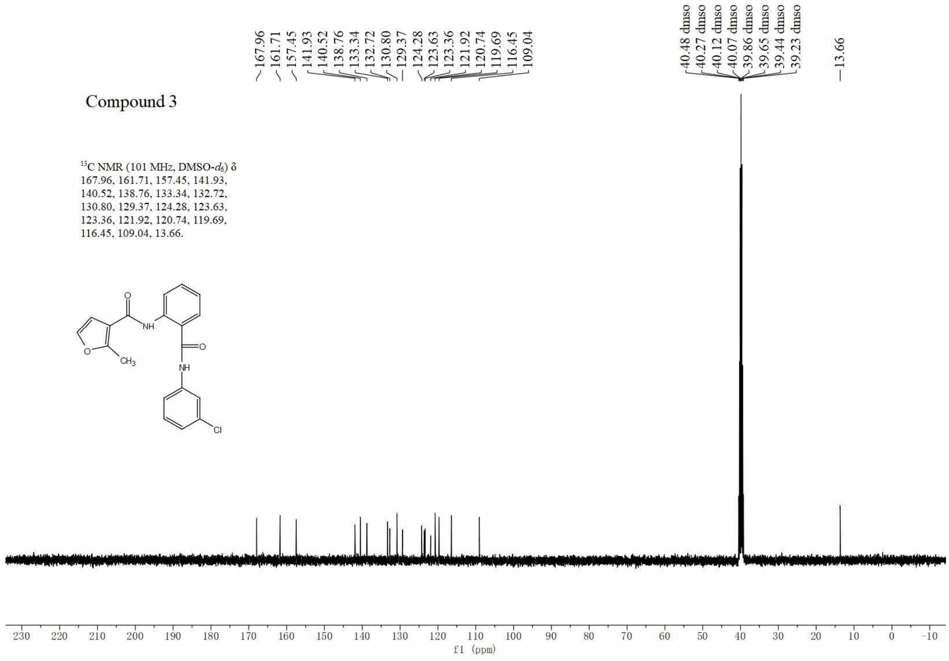

The ^{13}C NMR spectrogram of compound **3**

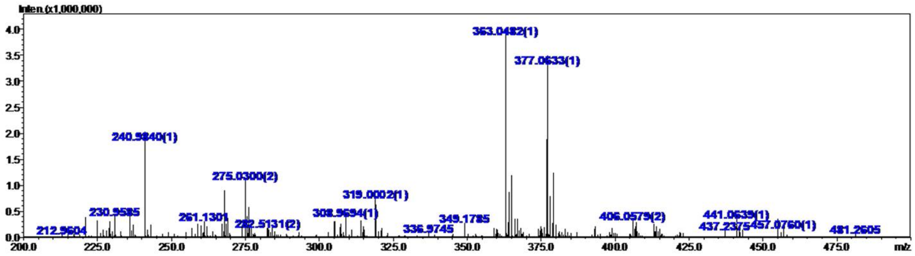

The HRMS of compound **3**

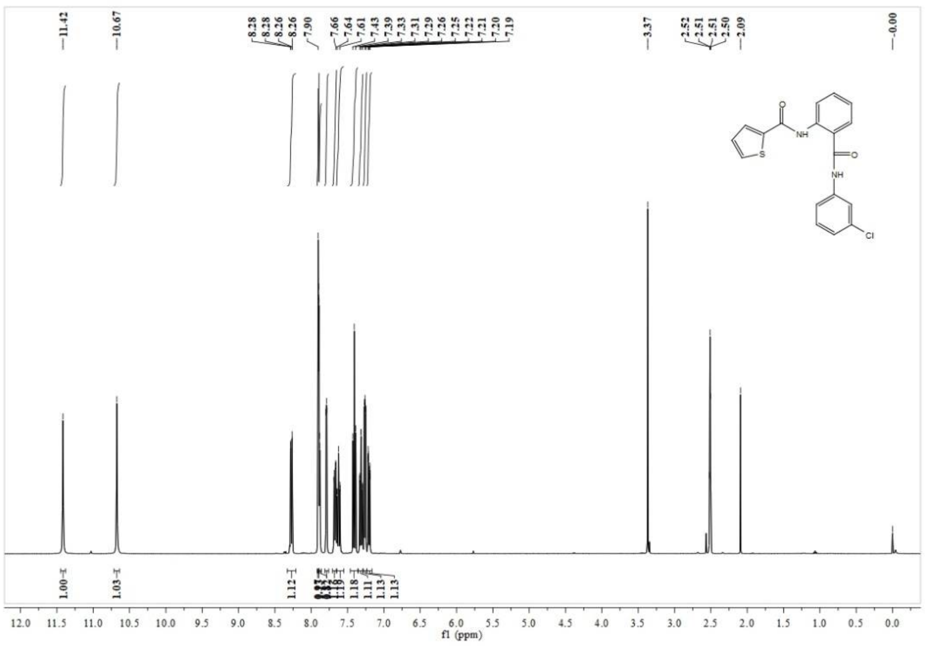

The ^{1}H NMR spectrogram of compound **4**

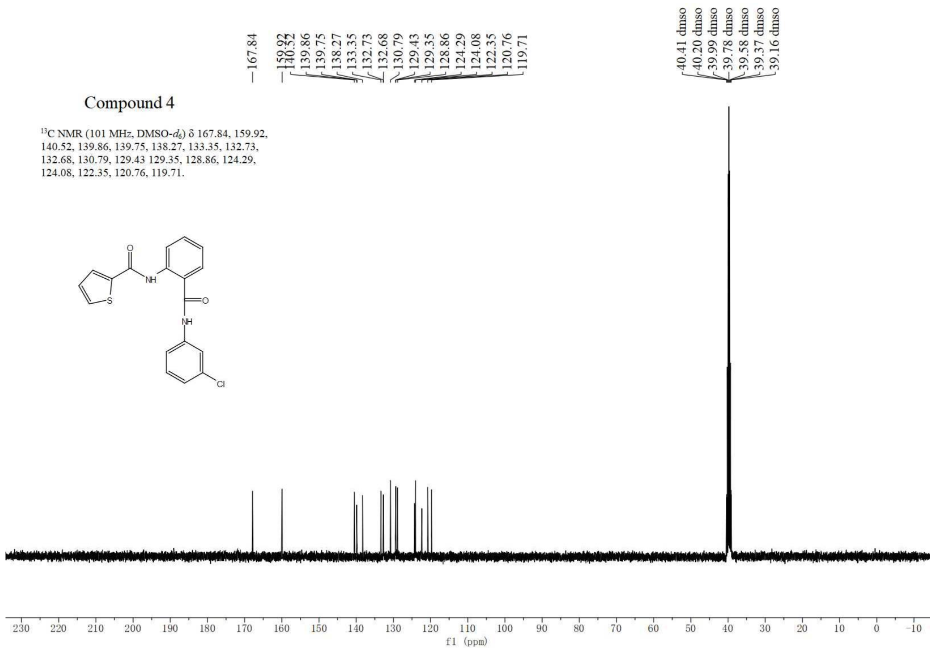

The ^{13}C NMR spectrogram of compound **4**

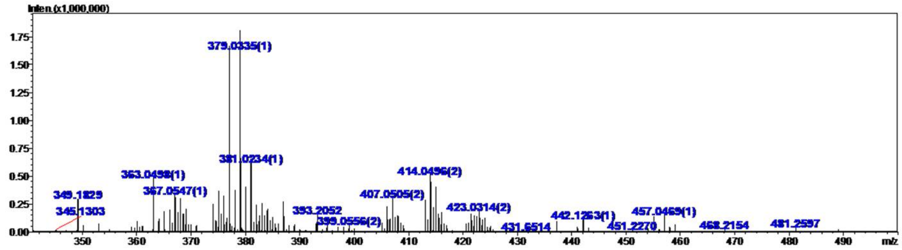

The HRMS of compound **4**

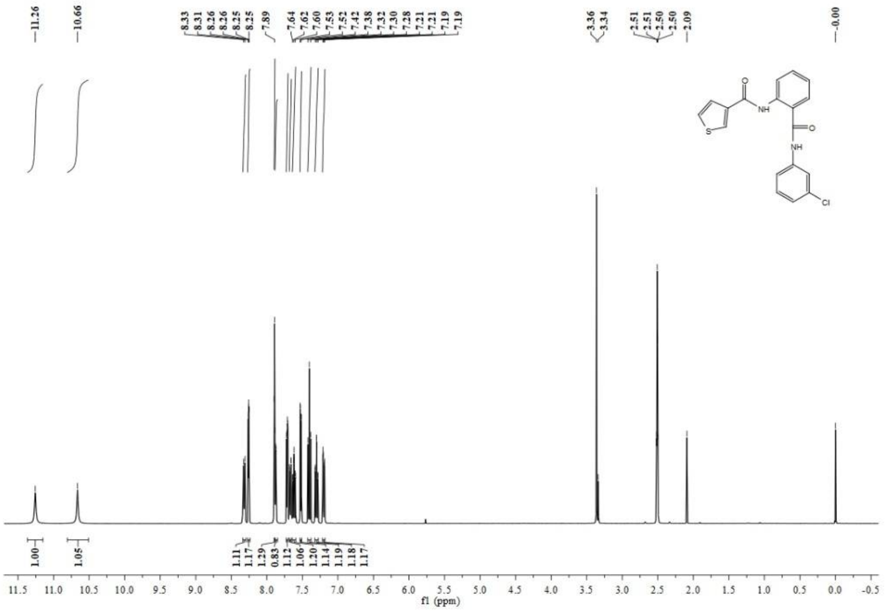

The ^{1}H NMR spectrogram of compound **5**

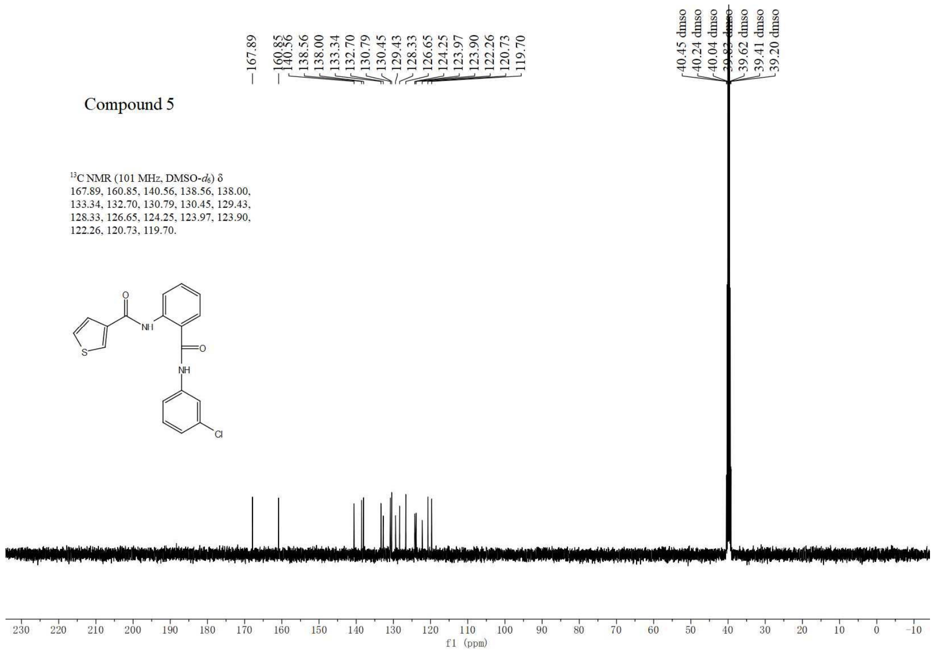

The ^{13}C NMR spectrogram of compound **5**

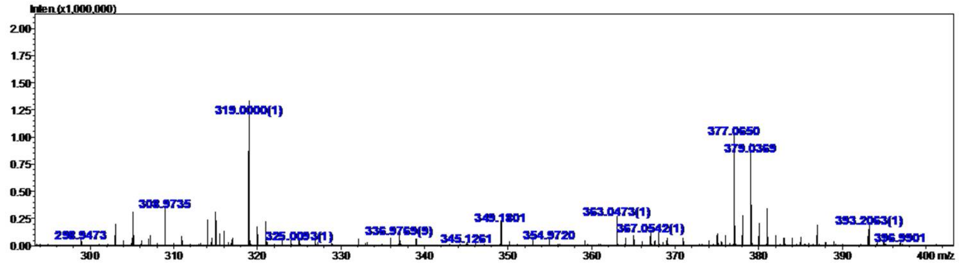

The HRMS of compound **5**

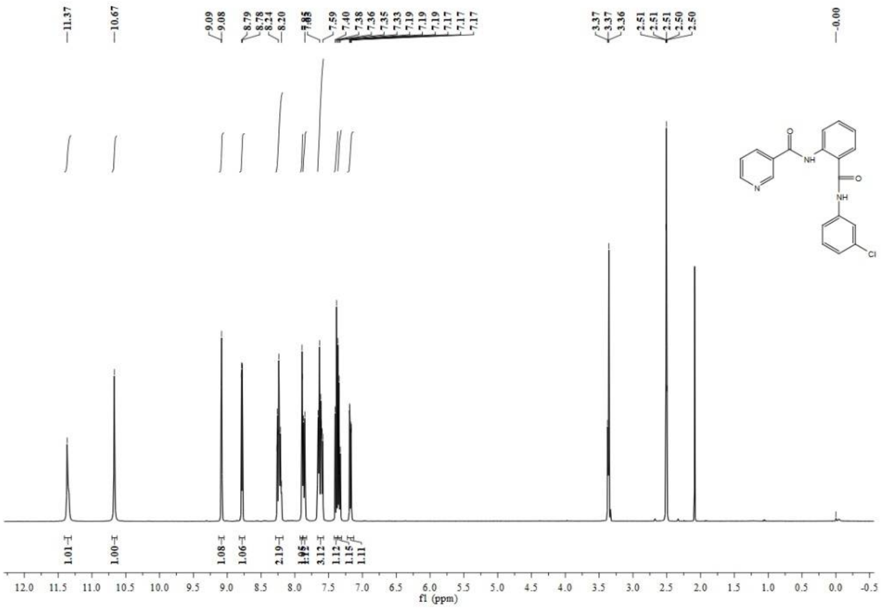

The ^{1}H NMR spectrogram of compound **6**

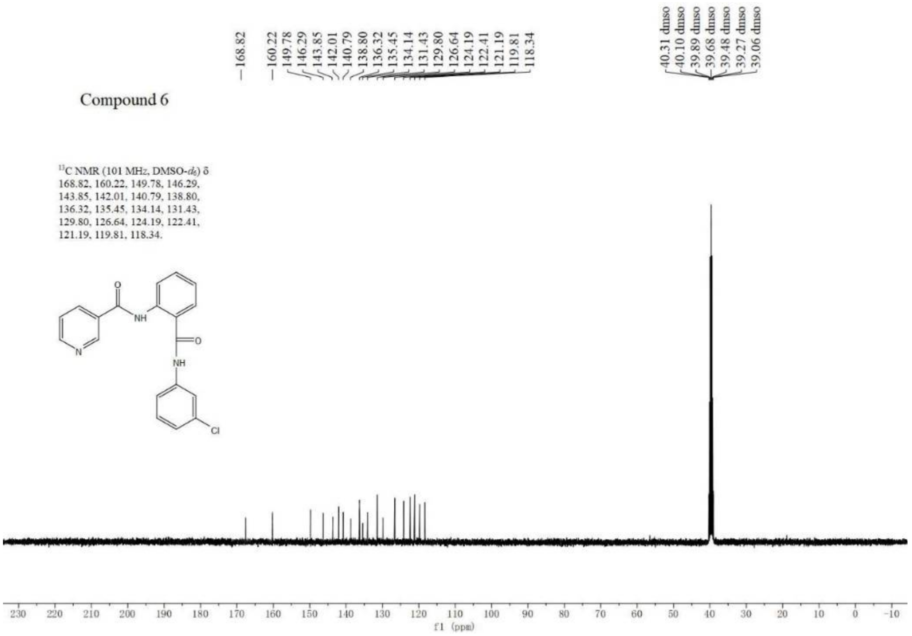

The ^{13}C NMR spectrogram of compound **6**

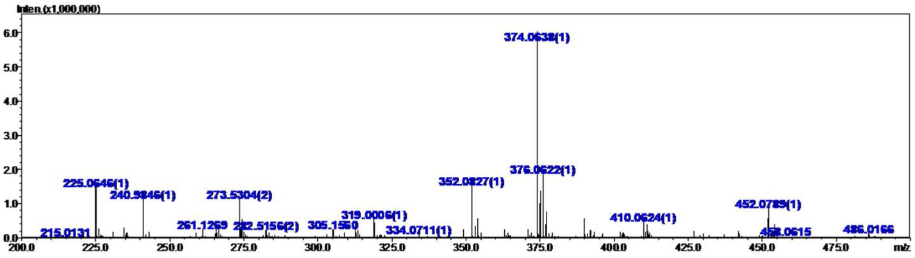

The HRMS of compound **6**

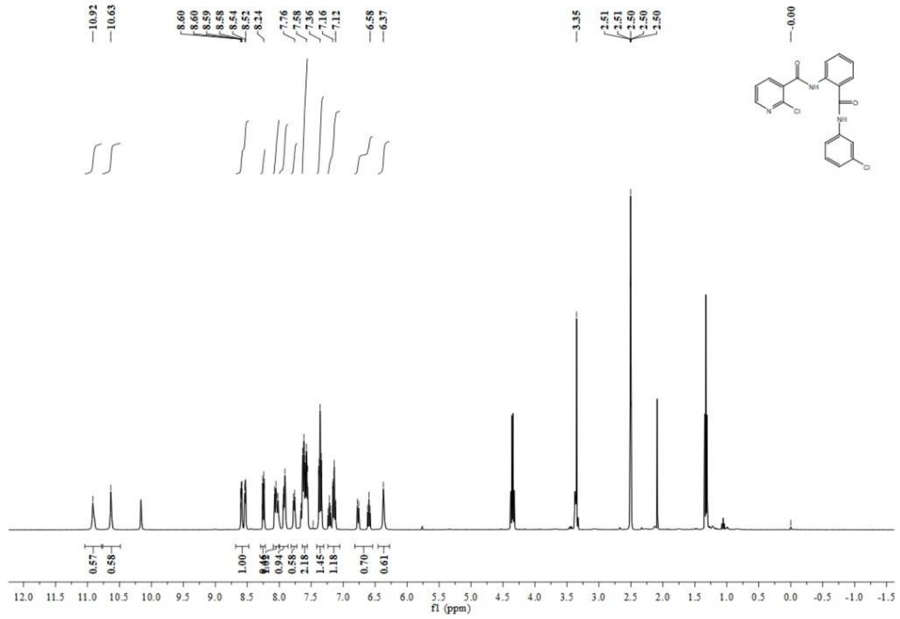

The ^{1}H NMR spectrogram of compound **7**

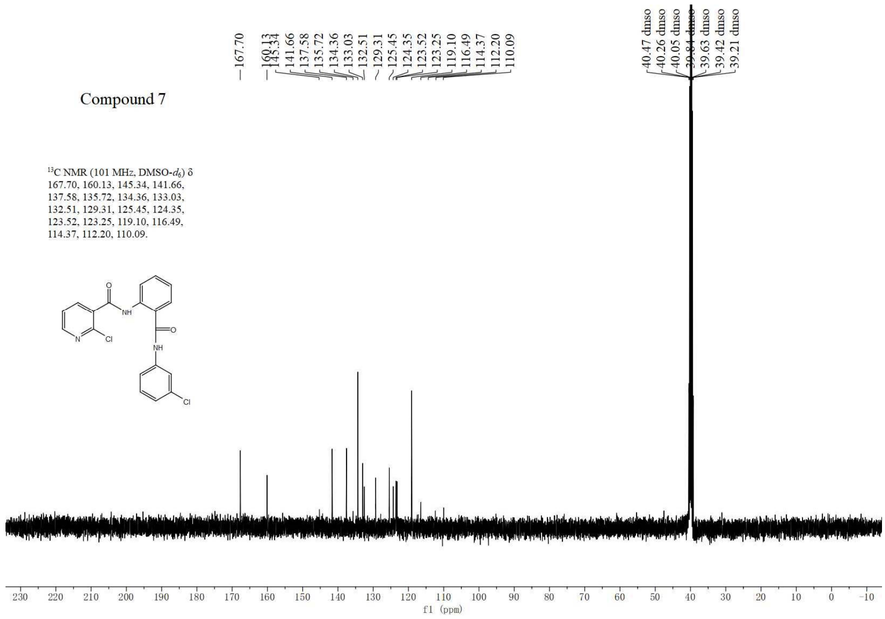

The ^{13}C NMR spectrogram of compound **7**

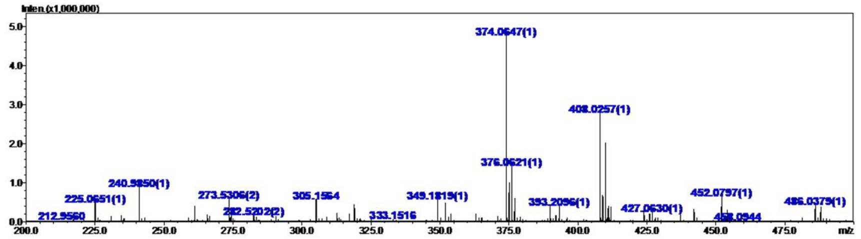

The HRMS of compound **7**

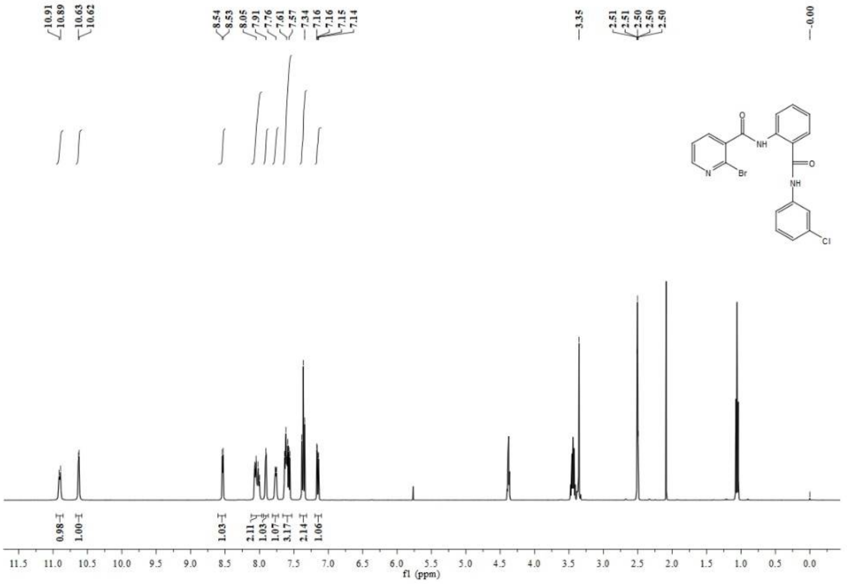

The ^{1}H NMR spectrogram of compound **8**

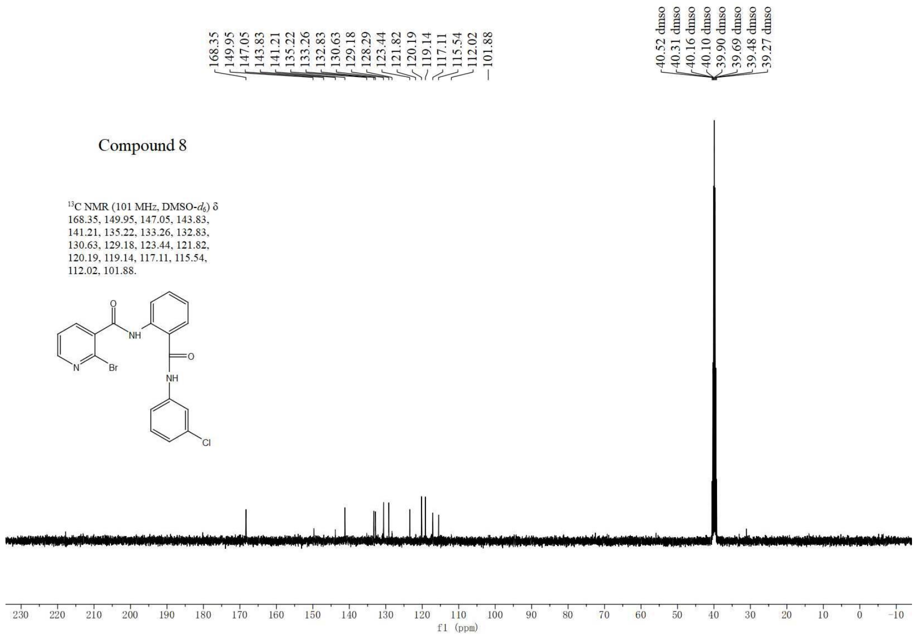

The ^{13}C NMR spectrogram of compound **8**

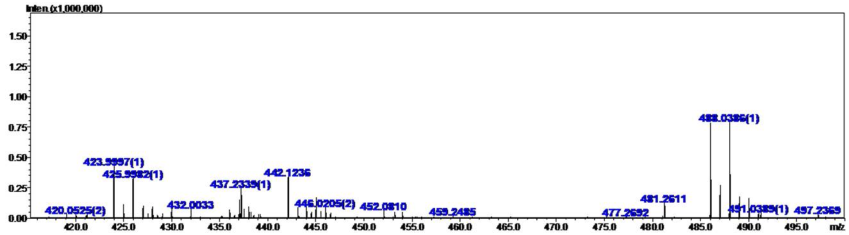

The HRMS of compound **8**

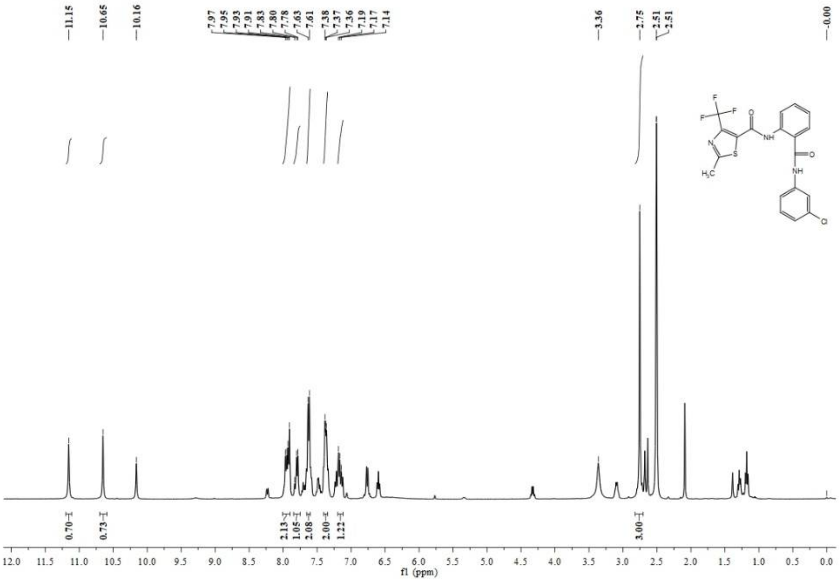

The ^{1}H NMR spectrogram of compound **9**

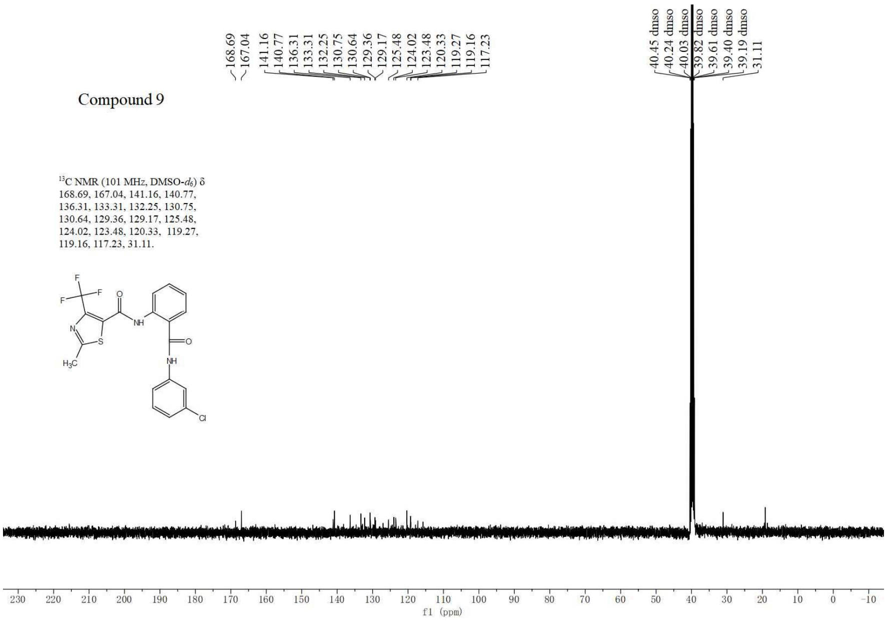

The ^{13}C NMR spectrogram of compound **9**

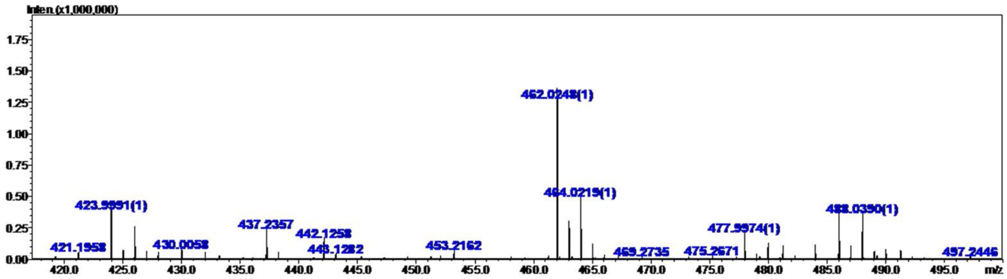

The HRMS of compound **9**

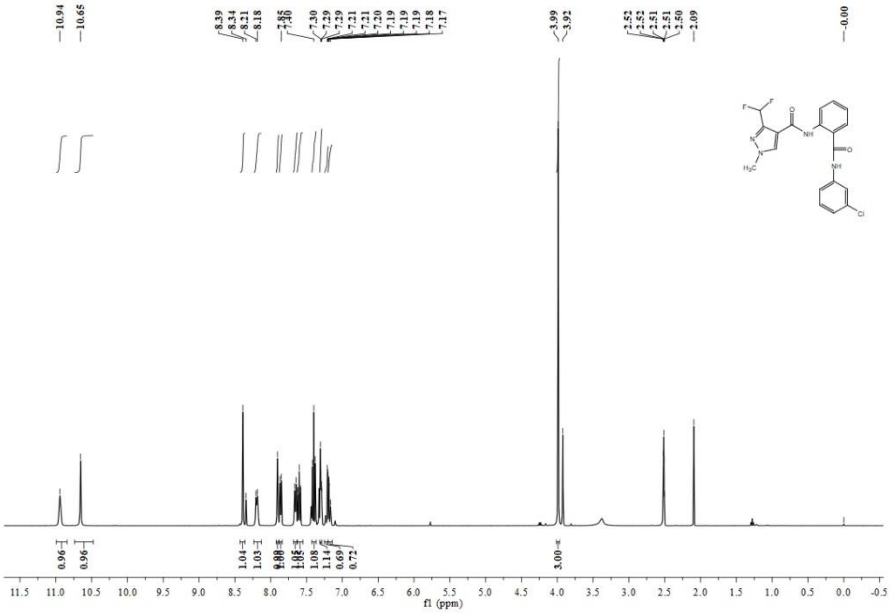

The ^{1}H NMR spectrogram of compound **10**

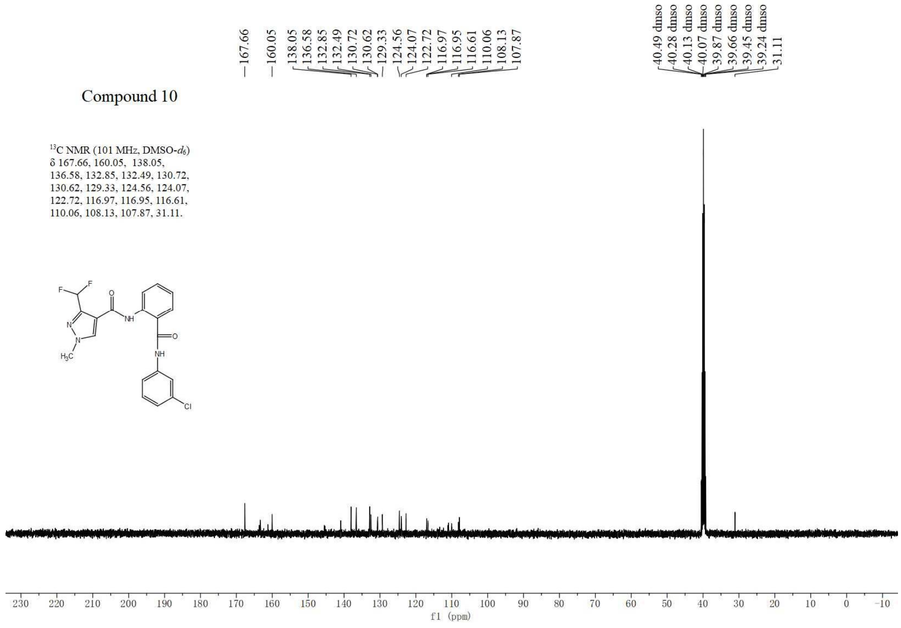

The ^{13}C NMR spectrogram of compound **10**

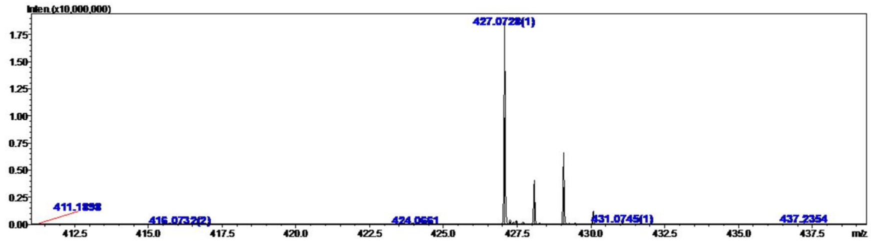

The HRMS of compound **10**

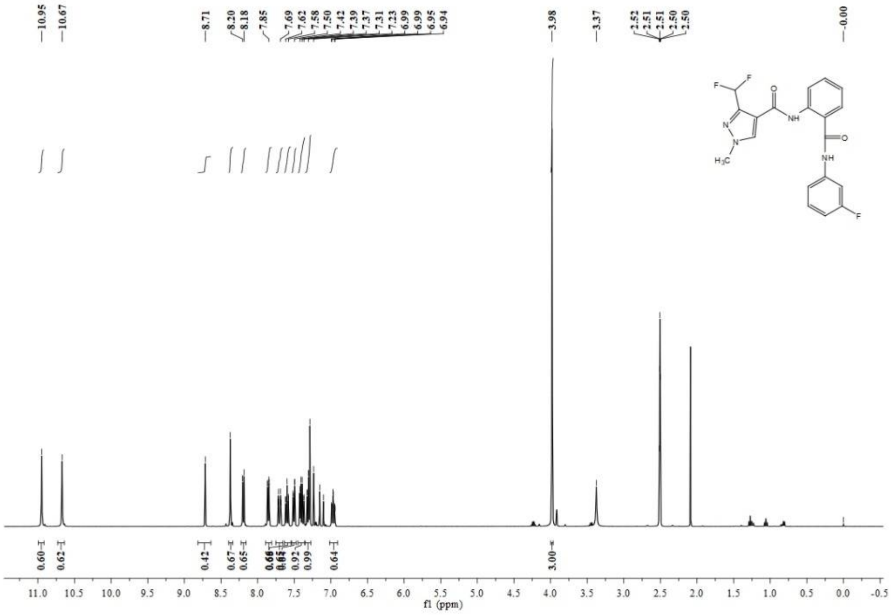

The ^{1}H NMR spectrogram of compound **11**

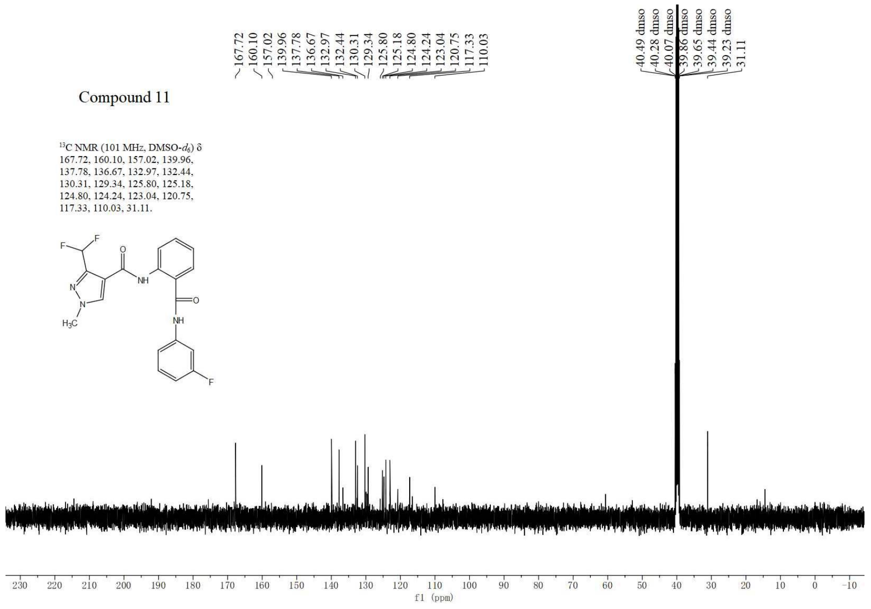

The ^{13}C NMR spectrogram of compound **11**

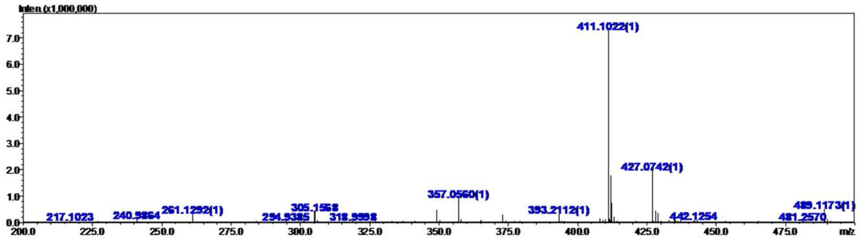

The HRMS of compound **11**

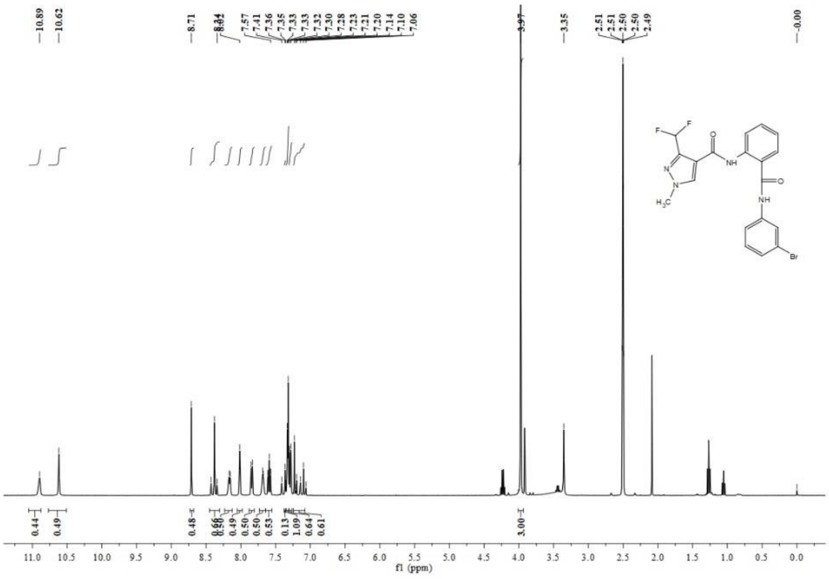

The ^{1}H NMR spectrogram of compound **12**

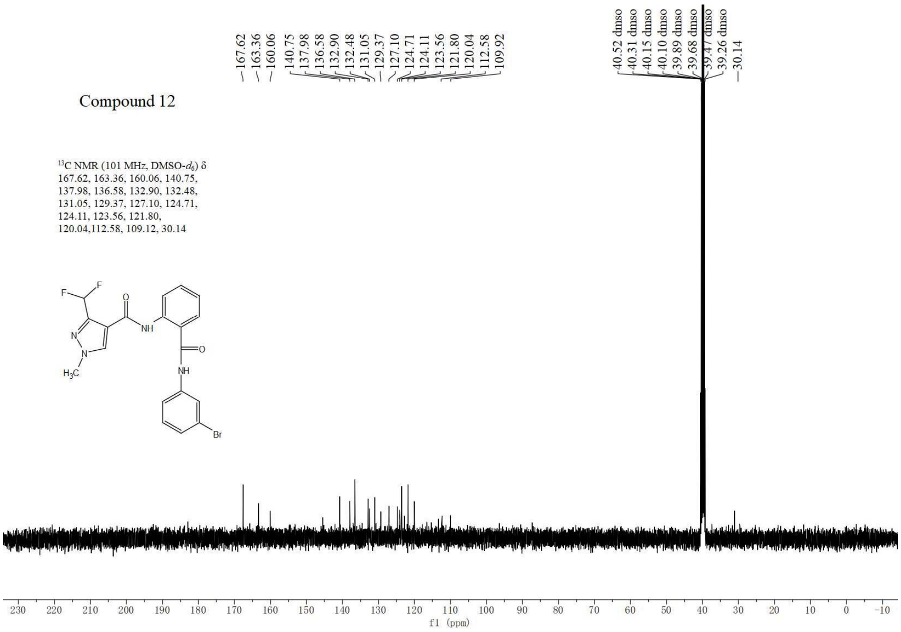

The ^{13}C NMR spectrogram of compound **12**

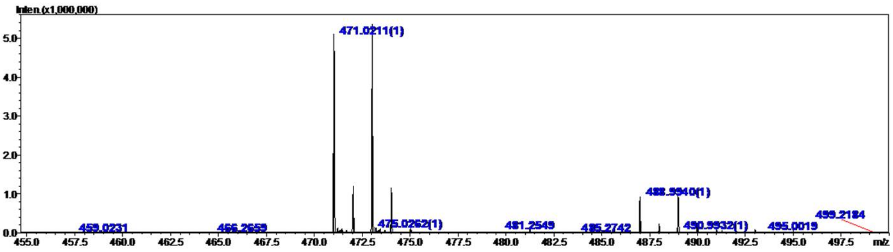

The HRMS of compound **12**

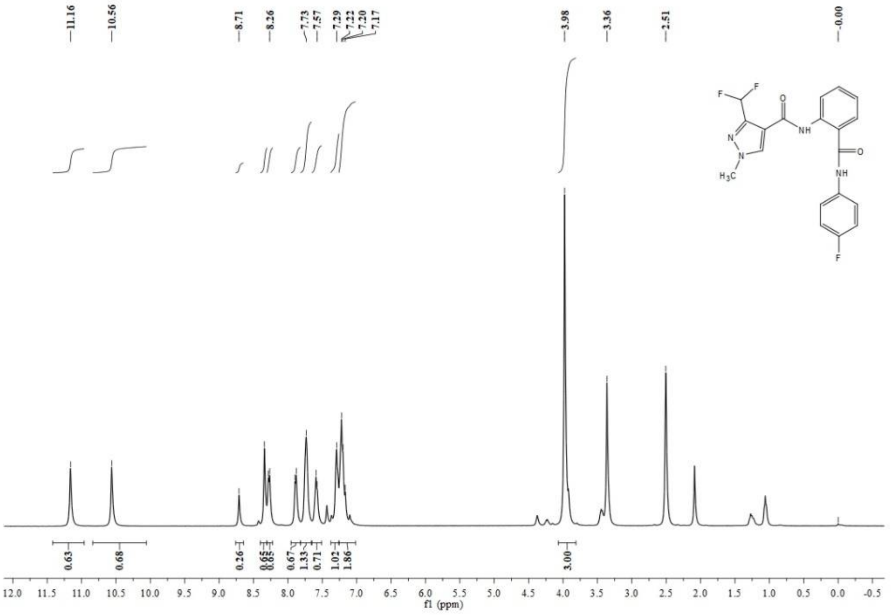

The ^{1}H NMR spectrogram of compound **13**

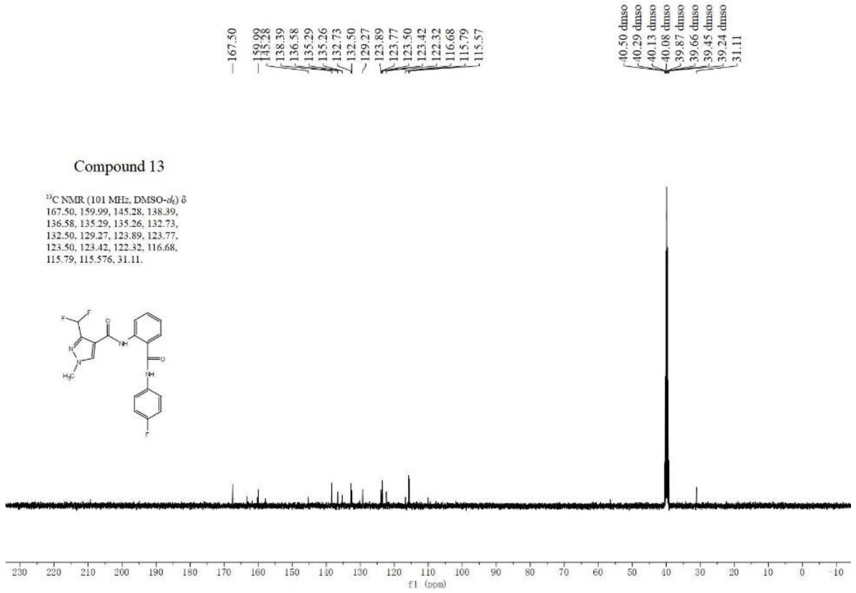

The ^{13}C NMR spectrogram of compound **13**

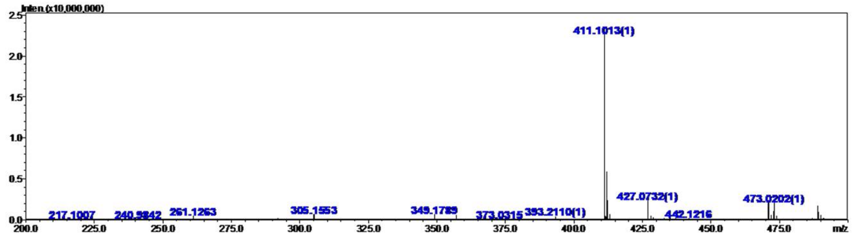

The HRMS of compound **13**

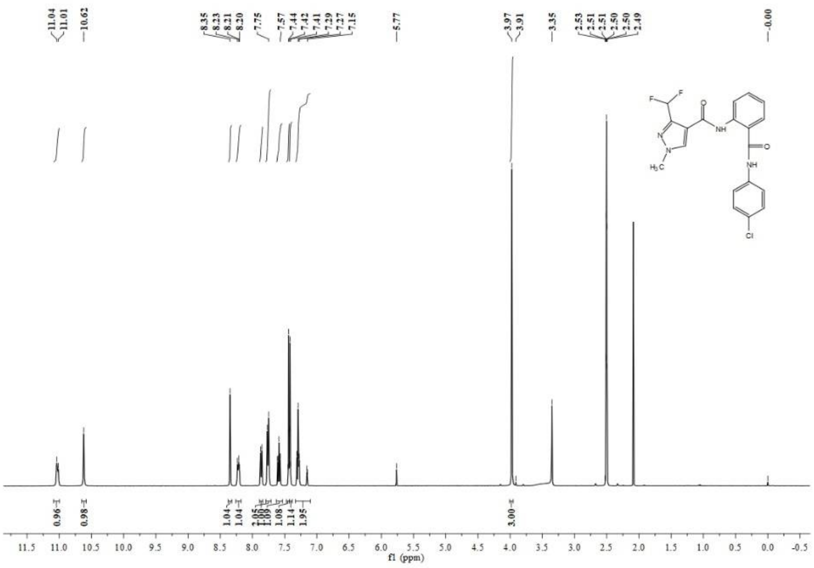

The ^{1}H NMR spectrogram of compound **14**

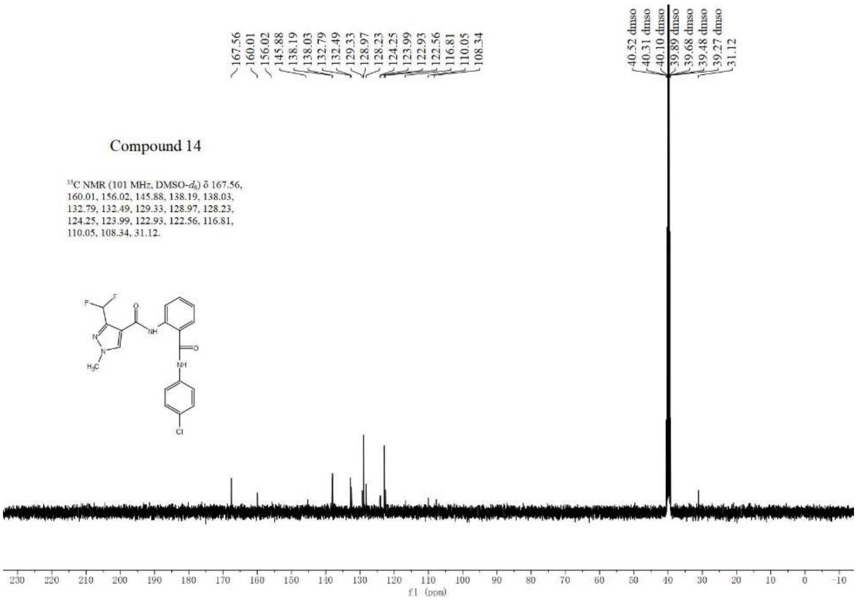

The ^{13}C NMR spectrogram of compound **14**

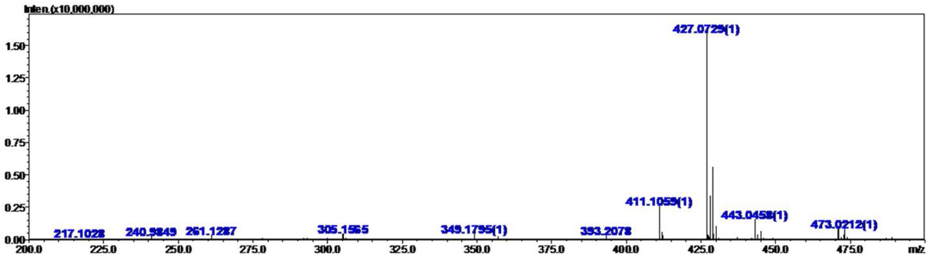

The HRMS of compound **14**

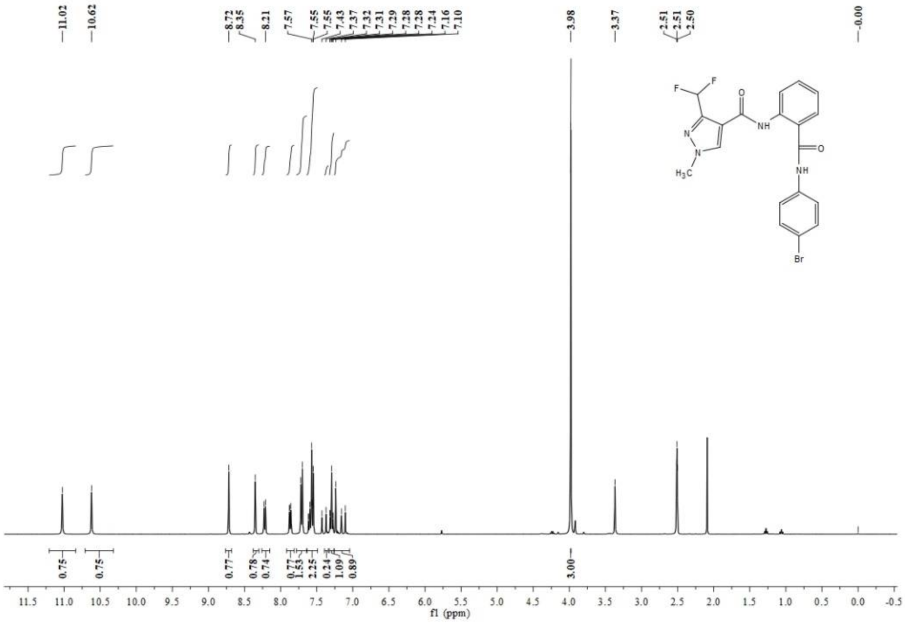

The ^{1}H NMR spectrogram of compound **15**

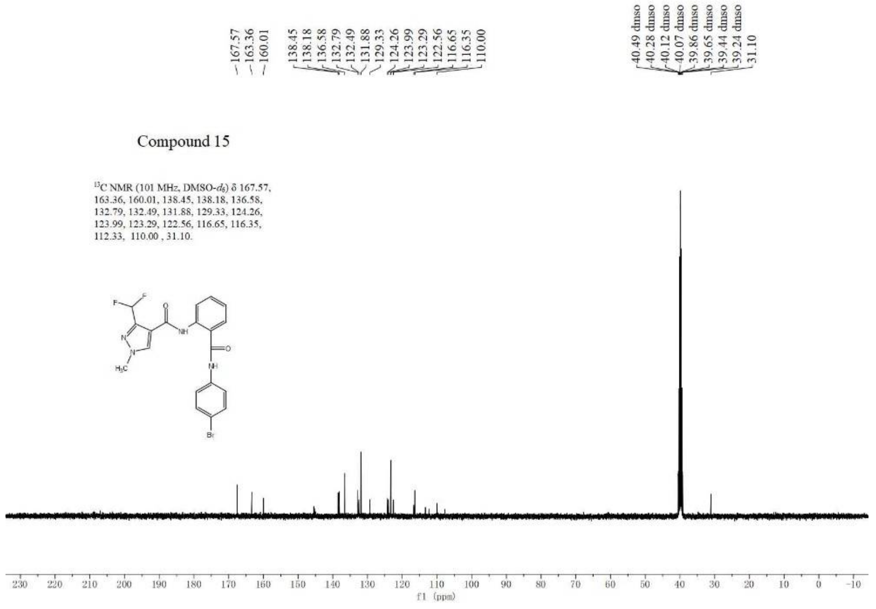

The ^{13}C NMR spectrogram of compound **15**

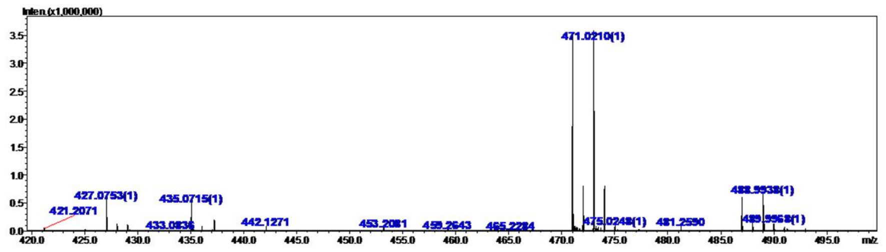

The HRMS of compound **15**

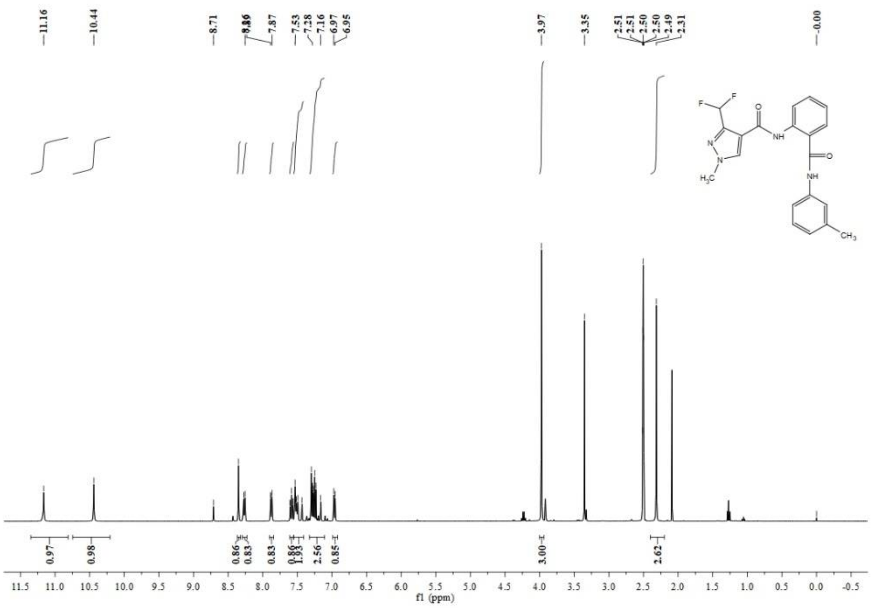

The ^{1}H NMR spectrogram of compound **16**

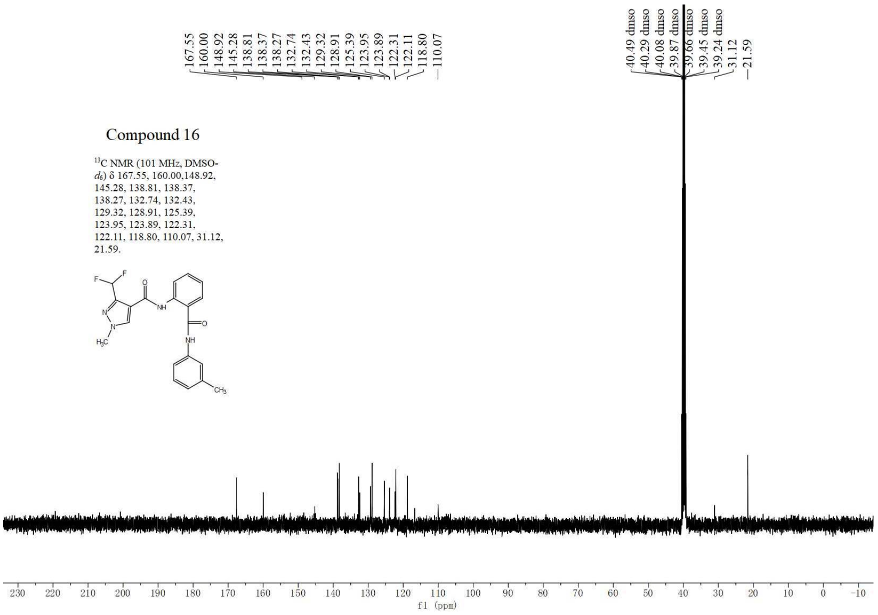

The ^{13}C NMR spectrogram of compound **16**

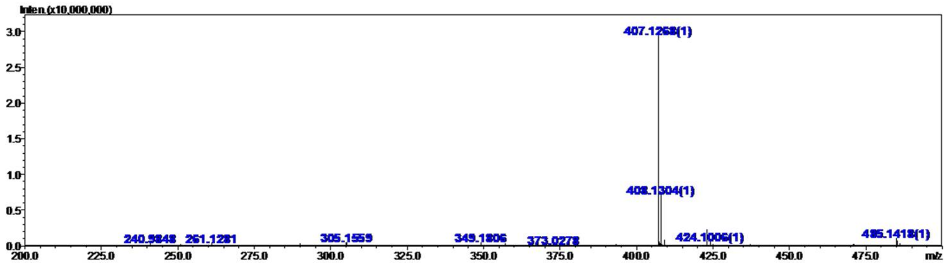

The HRMS of compound **16**

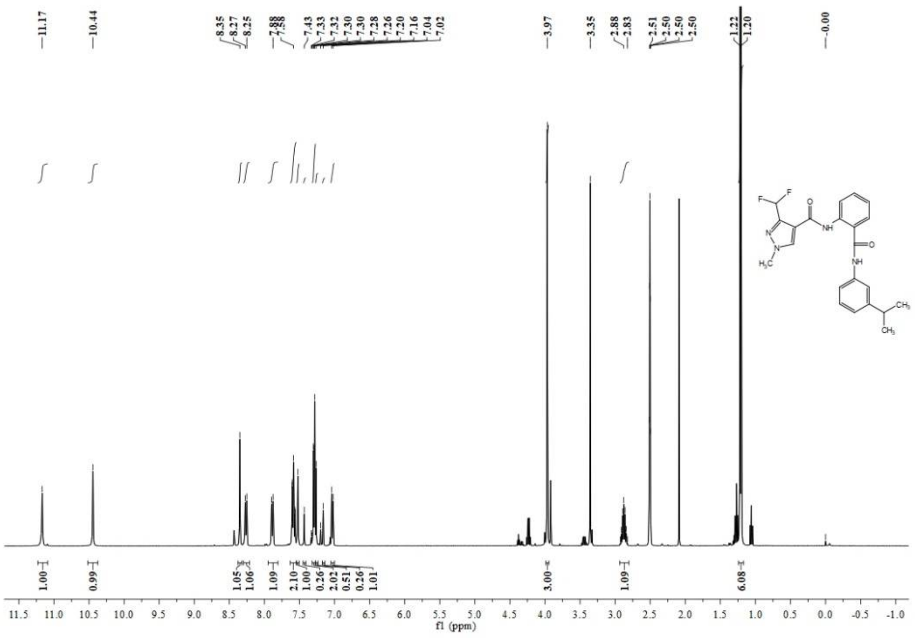

The ^{1}H NMR spectrogram of compound **17**

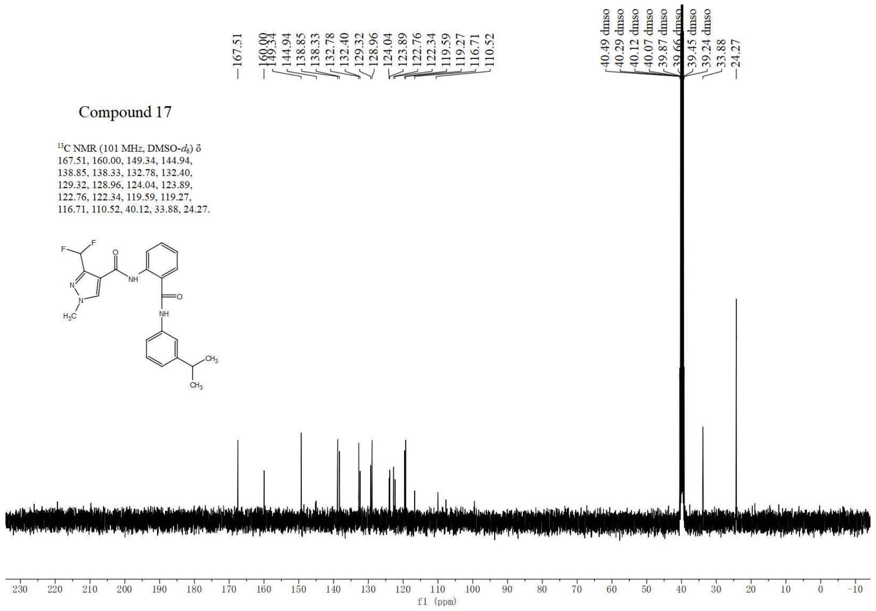

The ^{13}C NMR spectrogram of compound **17**

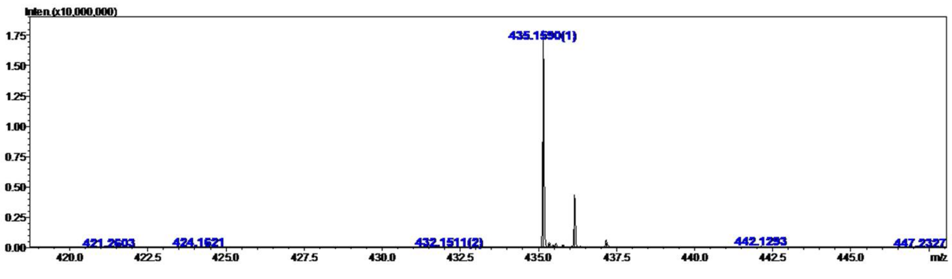

The HRMS of compound **17**

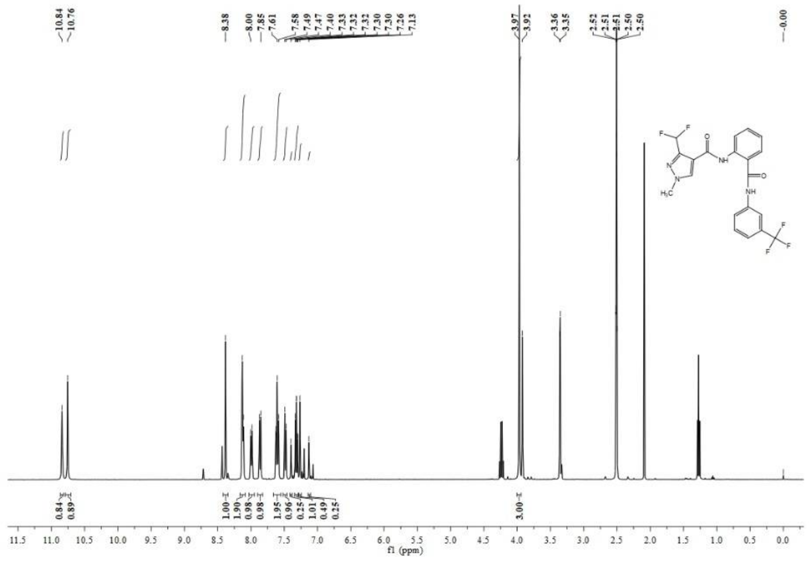

The ^{1}H NMR spectrogram of compound **18**

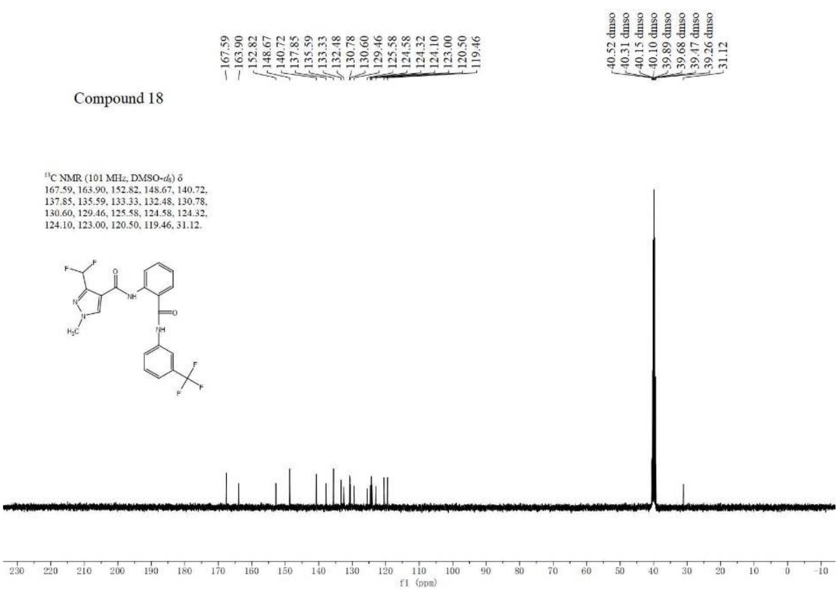

The ^{13}C NMR spectrogram of compound **18**

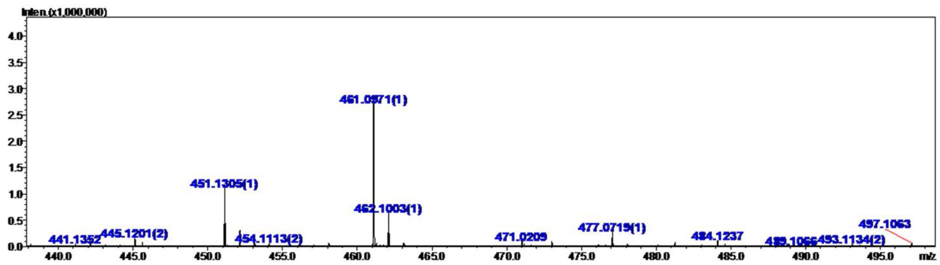

The HRMS of compound **18**

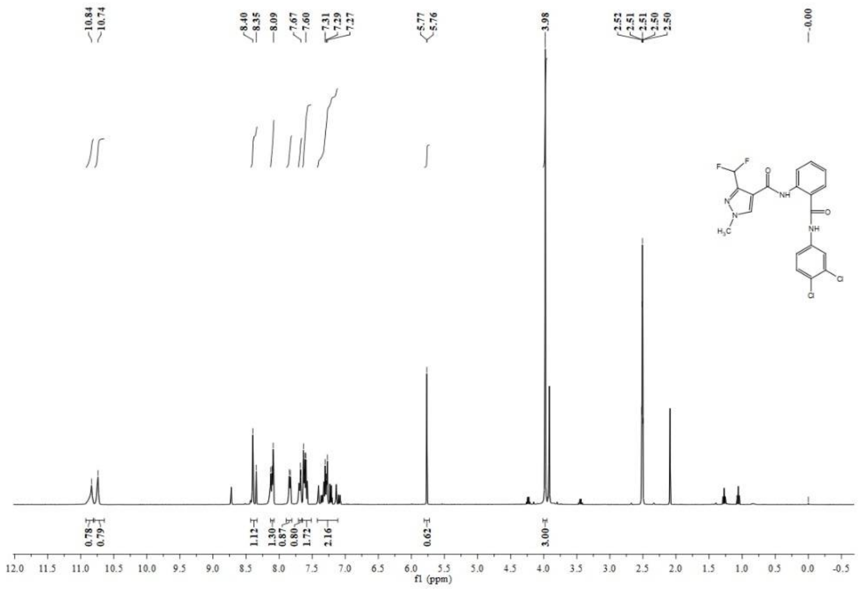

The ^{1}H NMR spectrogram of compound **19**

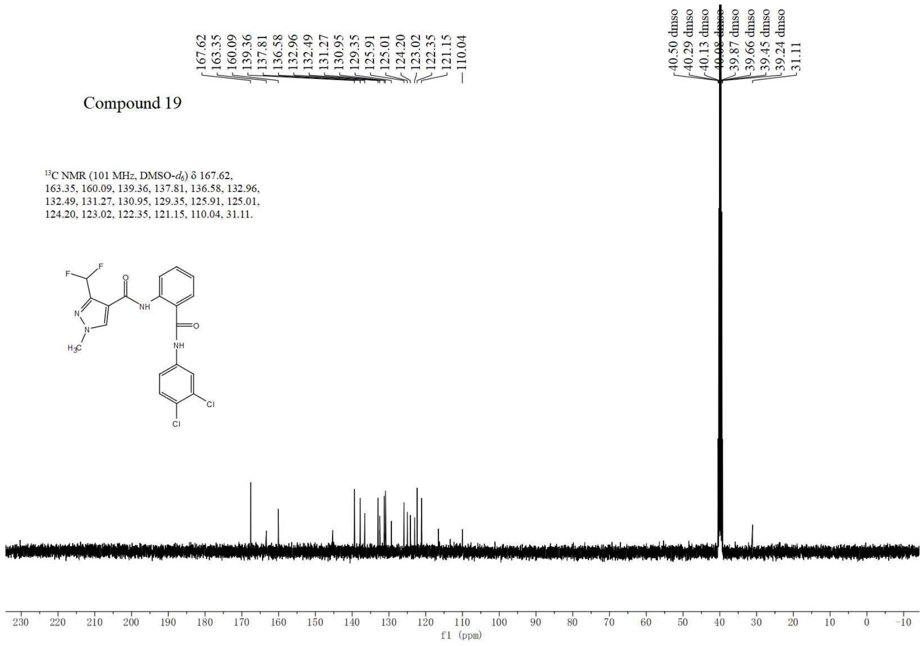

The ^{13}C NMR spectrogram of compound **19**

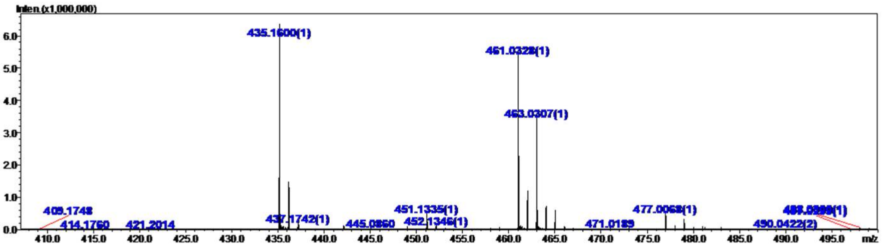

The HRMS of compound **19**

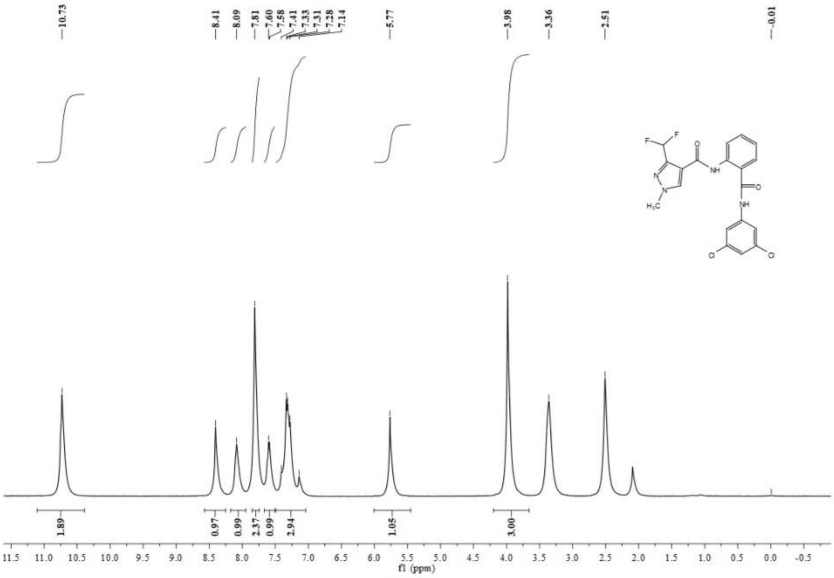

The ^{1}H NMR spectrogram of compound **20**

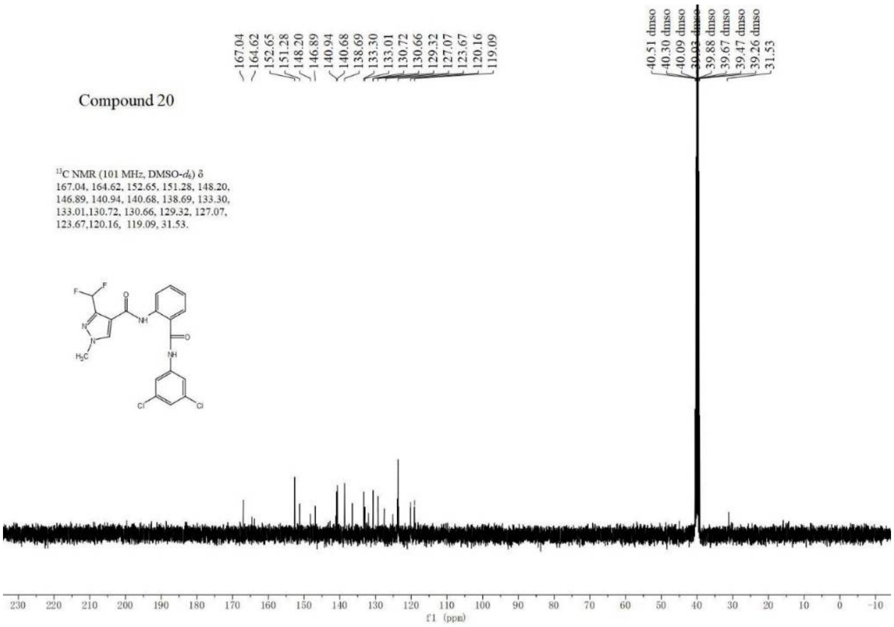

The ^{13}C NMR spectrogram of compound **20**

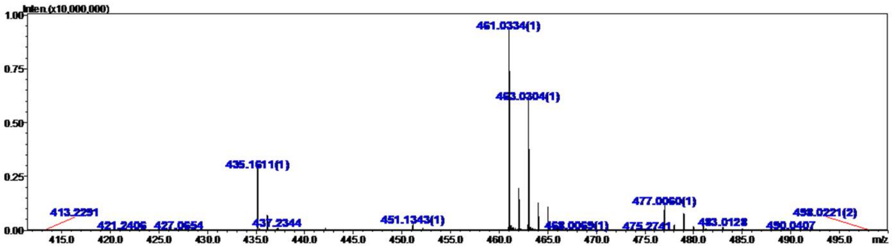

The HRMS of compound **20**
